# Estimated impact of revising the 13-valent pneumococcal conjugate vaccine schedule from 2+1 to 1+1 in England and Wales: A modelling study

**DOI:** 10.1371/journal.pmed.1002845

**Published:** 2019-07-03

**Authors:** Yoon Hong Choi, Nick Andrews, Elizabeth Miller

**Affiliations:** 1 Statistics, Modelling and Economics Department, Data and Analytical Sciences, National Infection Service, Public Health England, London, United Kingdom; 2 Immunisation and Countermeasures Division, National Infection Service, Public Health England, London, United Kingdom; The National Institute for Public Health and the Environment, NETHERLANDS

## Abstract

**Background:**

In October 2017, the United Kingdom Joint Committee on Vaccination and Immunisation (JCVI) recommended removal of one primary dose of the 13-valent pneumococcal conjugate vaccine (PCV13) from the existing 2+1 schedule (2, 4, 12 months). We conducted a mathematical modelling study to investigate the potential impact of a 1+1 (3, 12 month) schedule on invasive pneumococcal disease (IPD) and pneumococcal community-acquired pneumonia (CAP). Our results and those from a 1+1 immunogenicity study formed the key evidence reviewed by JCVI.

**Methods and findings:**

We developed age-structured, dynamic, deterministic models of pneumococcal transmission in England and Wales to describe the impact on IPD of 7-valent PCV (PCV7; introduced in 2006) and PCV13 (introduced in 2010). Key transmission and vaccine parameters were estimated by fitting to carriage data from 2001/2002 and post-PCV IPD data to 2015, using vaccine coverage, mixing patterns between ages, and population data. We considered various models to investigate potential reasons for the rapid increase in non-PCV13 (non-vaccine serotype [NVT]) IPD cases since 2014. After searching a large parameter space, 500 parameter sets were identified with a likelihood statistically close to the maximum and these used to predict future cases (median, prediction range from 500 parameter sets). Our findings indicated that the emergence of individual NVTs with higher virulence resulting from ongoing replacement was likely responsible; the NVT increase was predicted to plateau from 2020. Long-term simulation results suggest that changing to a 1+1 schedule would have little overall impact, as the small increase in vaccine-type IPD would be offset by a reduction in NVT IPD. Our results were robust to changes in vaccine assumptions in a sensitivity analysis. Under the base case scenario, a change to a 1+1 schedule in 2018 was predicted to produce 31 (6, 76) additional IPD cases over five years and 83 (−10, 242) additional pneumococcal-CAP cases, with together 8 (−2, 24) additional deaths, none in children under 15 years. Long-term continuation with the 2+1 schedule, or changing to a 1+1, was predicted to sustain current reductions in IPD cases in under-64-year-olds, but cases in 65+-year-olds would continue to increase because of the effects of an aging population. Limitations of our model include difficulty in fitting to past trends in NVT IPD in some age groups and inherent uncertainty about future NVT behaviour, sparse data for defining the mixing matrix in 65+-year-olds, and the methodological challenge of defining uncertainty on predictions.

**Conclusions:**

Our findings suggest that, with the current mature status of the PCV programme in England and Wales, removing one primary dose in the first year of life would have little impact on IPD or pneumococcal CAP cases or associated deaths at any age. A reduction in the number of priming doses would improve programmatic efficiency and facilitate the introduction of new vaccines by reducing the number of coadministered vaccines given at 2 and 4 months of age in the current UK schedule. Our findings should not be applied to other settings with different pneumococcal epidemiology or with immature programmes and poor herd immunity.

## Abstract 2

Using a pneumococcal transmission model, Yoon Hong Choi and colleagues estimate the impact of removing one of the two infant PCV doses given in England and Wales.

## Author summary

**Why was this study done?:**

- The pneumococcus is a bacterium of global importance that causes pneumonia, septicaemia, and meningitis with many associated deaths, particularly in young children and the elderly.
- Pneumococcal conjugate vaccines (PCVs) that protect against the development of pneumococcal disease in those who are vaccinated and also prevent transmission of the pneumococcus in the population, resulting in herd immunity, are highly effective but expensive.
- In populations with high PCV coverage and good herd immunity, overall PCV programmatic costs could potentially be reduced without much impact on pneumococcal disease by removing one of the two doses given in the first year of life, as the booster dose given in the second year of life is thought to be key for maintaining herd immunity.
- We investigated the likely impact of removing one of the two infant PCV doses given in the national immunisation programme in England and Wales using a pneumococcal transmission model.

**What did the researchers do and find?:**

- The model was fitted to past trends in the incidence of laboratory-confirmed pneumococcal cases of septicaemia and meningitis in England and Wales, both in infants who had received PCV vaccines and in older age groups who had indirectly benefitted through herd immunity.
- The fitted model was then used to predict the likely impact on pneumococcal disease if one of the two infant doses currently given at 2 and 4 months of age in England and Wales was replaced by a single PCV dose at 3 months of age.
- The results indicated that any increase in pneumococcal disease by dropping one infant PCV dose would be extremely small and that the current health gains from the PCV programme in England and Wales would be preserved.

**What do these findings mean?:**

- Our findings could help improve overall PCV programmatic efficiency by saving on vaccine purchase costs with little adverse impact on disease control.
- Based on this modelling study, the UK committee that makes recommendations on the national immunisation programme has already advised dropping one of the two infant doses in the UK PCV programme.
- Our model findings could be applied to other countries with a similar mature PCV programme and pneumococcal epidemiology to England and Wales.
- For countries in regions such as Africa, where pneumococcal disease rates are higher, PCVs have only recently been introduced, and their impact in vaccinated and older age groups has not yet been fully elucidated. While our modelling results cannot be directly translated into policy in such regions, models such as ours could help in understanding epidemiological trends after PCV introduction and in identifying optimal vaccination schedules.

## Introduction

The pneumococcus is a ubiquitous bacterial pathogen of global importance. The organism is carried asymptomatically in the nasopharynx but can spread to the lower respiratory tract, resulting in pneumonia, or, more rarely, invade the blood stream, causing septicaemia or meningitis. All age groups are affected with morbidity and mortality, particularly high in young children and the elderly. The development of highly effective pneumococcal conjugate vaccines (PCVs), in which the capsular polysaccharides of the serotypes most commonly causing invasive disease are conjugated to a protein carrier, has been a major advance [1]. Paediatric PCV programmes have greatly reduced the incidence of vaccine-type invasive disease in vaccinated children and in older unvaccinated groups, the latter through reduction in carriage prevalence of vaccine serotypes and the generation of herd immunity. The World Health Organisation now recommends the inclusion of PCVs in childhood immunisation programmes worldwide [2].

The first generation of PCVs were 7 valent and initially licensed as a three-dose priming schedule in infants, with a booster in the second year of life. To reduce overall costs and improve programmatic efficiency, in 2006 the UK became the first country to use a reduced priming schedule for the 7-valent pneumococcal conjugate vaccine (PCV7), giving just two doses at 2 and 4 months of age, followed by a 12 month booster (2+1). This decision was based on evidence from a pivotal immunogenicity study [3] supported by predictions from a dynamic transmission model that simulated the impact of PCV7 on carriage and translated this into invasive pneumococcal disease (IPD) by using information on the invasiveness potential of PCV7 and non-PCV7 serotypes [4,5]. Post-licensure surveillance in England and Wales [6] confirmed the predicted reduction in IPD caused by PCV7 serotypes in vaccinated children and the unvaccinated population, resulting in the adoption of 2+1 schedules by many other countries [7]. In 2010, the UK in common with other countries replaced PCV7 with the 13-valent vaccine (PCV13), covering six additional serotypes, including 3, 7F, and 19A, whose prevalence had increased in carriage and IPD post-PCV7 [6,8]. Comparison of the prevalence of non-PCV13 serotypes in carriage with that in IPD showed that most had a low propensity to cause invasive disease when carried. This suggested that even though non-PCV13 serotypes might increase in carriage post-PCV13, there would nevertheless be an overall reduction in IPD [8]. This conclusion was supported by predictions from a transmission model, which suggested that even with the worst-case scenario of complete replacement of PCV13 by non-PCV13 serotypes in carriage, there was still likely to be an overall reduction in IPD due to the lower invasiveness potential of non-PCV13 serotypes [9].

This prediction was confirmed during the first 4 years of the PCV13 programme in England and Wales with a 69% reduction in IPD due to the additional serotypes covered by PCV13 and a further 89% reduction in the PCV7 serotypes compared with the pre-PCV13 baseline [10]. Serotype replacement was modest, with only a 25% increase in non-PCV13 IPD, resulting in a 38% reduction in all IPD compared with pre-PCV13 levels. The success of the 2+1 schedule in reducing vaccine-type IPD in all age groups raised the question of whether, given the mature status of the UK programme, herd immunity could be maintained and programmatic efficiency further improved if one of the priming doses was dropped (1+1 schedule) [7,11]. A pivotal immunogenicity study that compared post-primary and post-booster responses after 1+1 (3 and 12 months) and 2+1 PCV13 schedules showed that although antibody levels achieved after one priming dose were inferior to those after two, post-booster responses were similar for most serotypes [7]. Assuming the immunogenicity results could be extrapolated to effectiveness against carriage, then a 1+1 schedule should provide similar protection against carriage post-booster but poorer protection post-primary than a 2+1 schedule.

The potential herd immunity effects of reducing protection in infants, in whom carriage prevalence is highest, was amenable to investigation by modelling. However, while the PCV13 transmission model fitted IPD trends up to 2013/2014, there was a rapid increase in non-PCV13 IPD cases beginning in 2014/2015 in England and Wales that was greater than predicted by the model, largely negating the further reduction in vaccine serotypes seen after PCV13 introduction [12]. In order to use the model to investigate the impact of a change to a 1+1 schedule, we first investigated potential scenarios that might have generated the observed increase in non-PCV13 IPD.

## Methods

### Serotype groupings in the model

We used an age-structured, deterministic model structure to describe the pneumococcal transmission dynamics in England and Wales, as developed by Choi and colleagues [9]. The model has three serotype groupings broadly corresponding to serotypes covered by PCV7, PCV13, or non-vaccine serotypes (NVTs), but with some modifications. PCV7 covers serotypes (STs) 4, 6B, 9V, 14, 18C, 19F, and 23F, with an additional six STs (1, 3, 5, 6A, 7F, and 19A) covered by PCV13. PCV acts by reducing carriage of the serotypes covered by the vaccine and, because some degree of competition in carriage between serotype groupings is assumed in the model, this results in an increase in the carriage prevalence of NVTs. This assumption did not hold for ST1, whose incidence fell progressively both before and after PCV7 introduction [6]. It was therefore excluded from both the PCV7 [5] and the subsequent PCV13 model [9]. Because ST1 declined further to very low levels in all age groups post-PCV13 [12], its exclusion will have little impact on future IPD incidence. PCV13 has not been shown to be effective against ST3 in the UK, and its incidence has increased progressively since 2013/2014 [12], thus behaving more like an NVT. In the model, serotypes are therefore stratified according to the following groupings: VT1 (PCV7), VT2 (the additional serotypes covered by PCV13 but without ST1 and ST3), and NVTs (non-PCV13 plus ST3).

### Data

As in previous modelling work [5,9], key model parameters governing transmission and competition between serotype groupings were derived by fitting to epidemiological data on carriage and IPD from the pre-PCV7 to post-PCV13 period (available in S1 Data), taking into account the changes in population structure in England and Wales over the period, the contact patterns between age groups, and age-specific PCV7 and PCV13 coverage data.

### Carriage prevalence and IPD incidence

**Fig 1:**
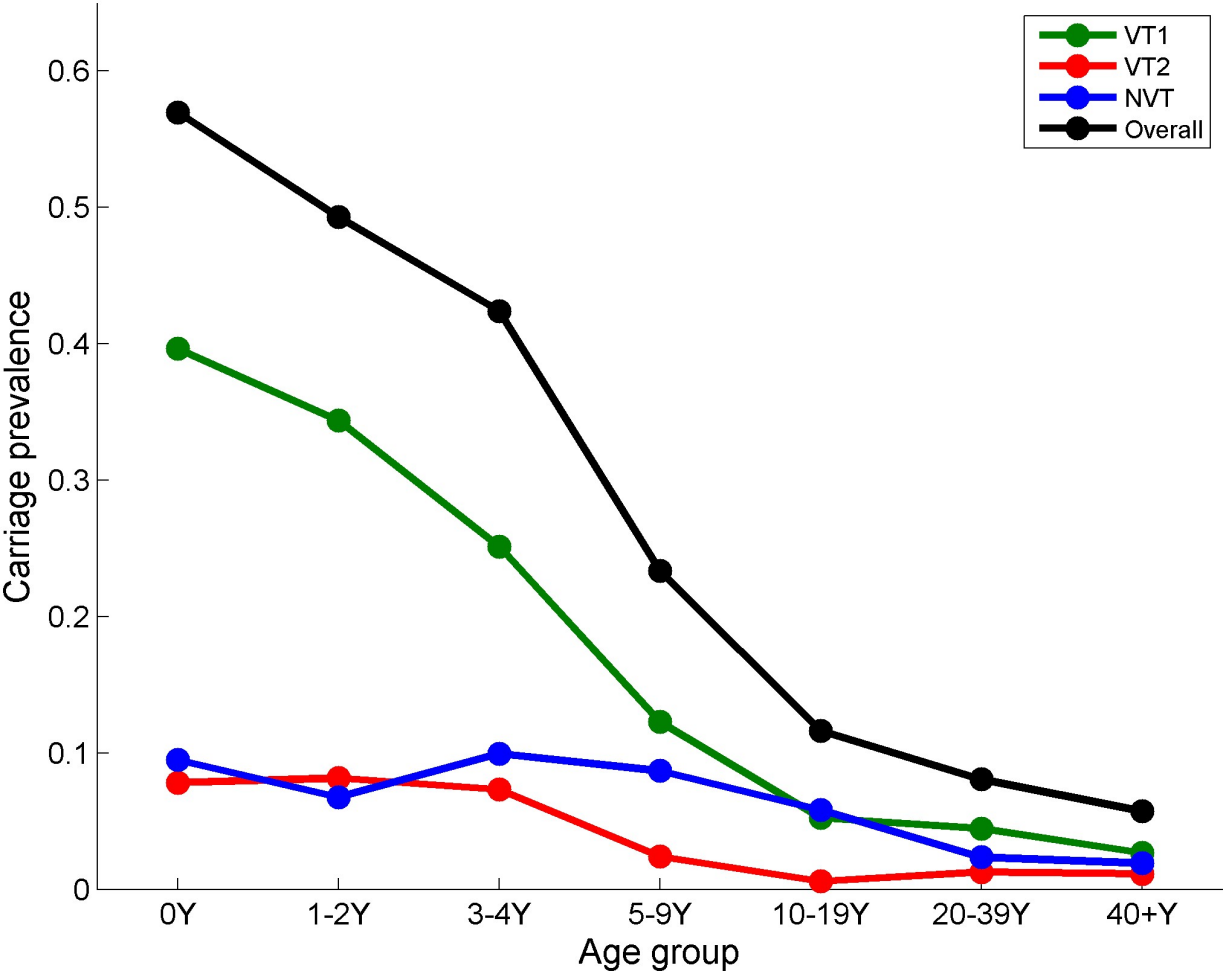
Carriage prevalence by seven age groups and three serotype groupings from the longitudinal carriage swab data collected in 2001/2002 (between 1 October 2001 and 14 August 2002) in England [13]. NVT, non-vaccine serotype group (non-PCV13 serotypes and serotype 3); PCV7, 7-valent pneumococcal conjugate vaccine; PCV13, 13-valent pneumococcal conjugate vaccine; VT1, vaccine serotype group 1 (PCV7 serotypes); VT2, vaccine serotype group 2 (serotypes only in PCV13 not in PCV7 excluding 1 and 3).

The pre-PCV7 carriage prevalence in seven age groups (under 1, 1–2, 3–4, 5–9, 10–19, 20–39, and 40+ years) and the three serotype groupings was obtained from a longitudinal family study in which 3,869 nasopharyngeal swabs were taken from 489 individuals who were swabbed monthly over 10 months between 1 October 2001 and 14 August 2002 in England. Carriage prevalence was highest in infants under one year of age (57% across all serotypes in this age group) (Fig 1). These samples were originally cultured for *Streptococcus pneumoniae* and serotyped by conventional methods [13]. Subsequently, a subset of samples from families in which all members provided at least five sequential swabs was retested by microarray-based molecular serotyping methods to detect episodes of multiple serotype carriage [14].

The national enhanced surveillance data set of serotyped IPD cases collated by Public Health England (PHE) for epidemiological years (from 1 July to 30 June of the subsequent calendar year) between 2000/2001 and 2015/2016 was used for model fitting, after adjustment for cases with missing age or serotype information, and from 2000/2001 to 2008/2009, with correction for the upward trend in all reported IPD resulting from improved surveillance sensitivity up to 2009/2010 [12]. The adjusted annual average number of IPD cases from 2000/2001 to 2005/2006 was used for the pre-PCV7 baseline incidence.

### Population structure and contact patterns between age groups

The annual population size by each yearly age from the pre-PCV7 to the post-PCV13 period was obtained from census data and the estimated demographic changes out to 2030 from the Office of National Statistics [15]. The contact pattern between age groups was derived from the POLYMOD survey conducted in the UK in 2006 [16], supplemented by an additional contact survey among infants under one year [17]. The contact matrix for transmission of carried pneumococci between and within the seven age groups was generated by combining these two contact surveys, with adjustment for the changes in the annual population size in England and Wales between 2005 and 2030.

### PCV coverage data

Vaccine coverage by dose, monthly birth cohort, and calendar month in the PCV7 catch-up and the routine 2+1 PCV7 programme up to 2008 was obtained from the General Practice Research database, as previously described [5]. Thereafter, coverage for the second priming dose and the booster dose was obtained from annual national coverage data, in which coverage for the second dose varied between 91%–93% and for the booster dose increased from 73% in 2008 to around 92% in subsequent years [18]. National coverage data for the first dose are not available, and so coverage was assumed to be 95% with all those receiving a second dose also receiving a first dose. The proportions receiving the priming and booster doses by month of age were obtained from the vaccination histories of children with non-PCV–type IPD in England and Wales who were followed up as controls for estimation of vaccine effectiveness [19].

### Model structure: Transition between model compartments

**Fig 2:**
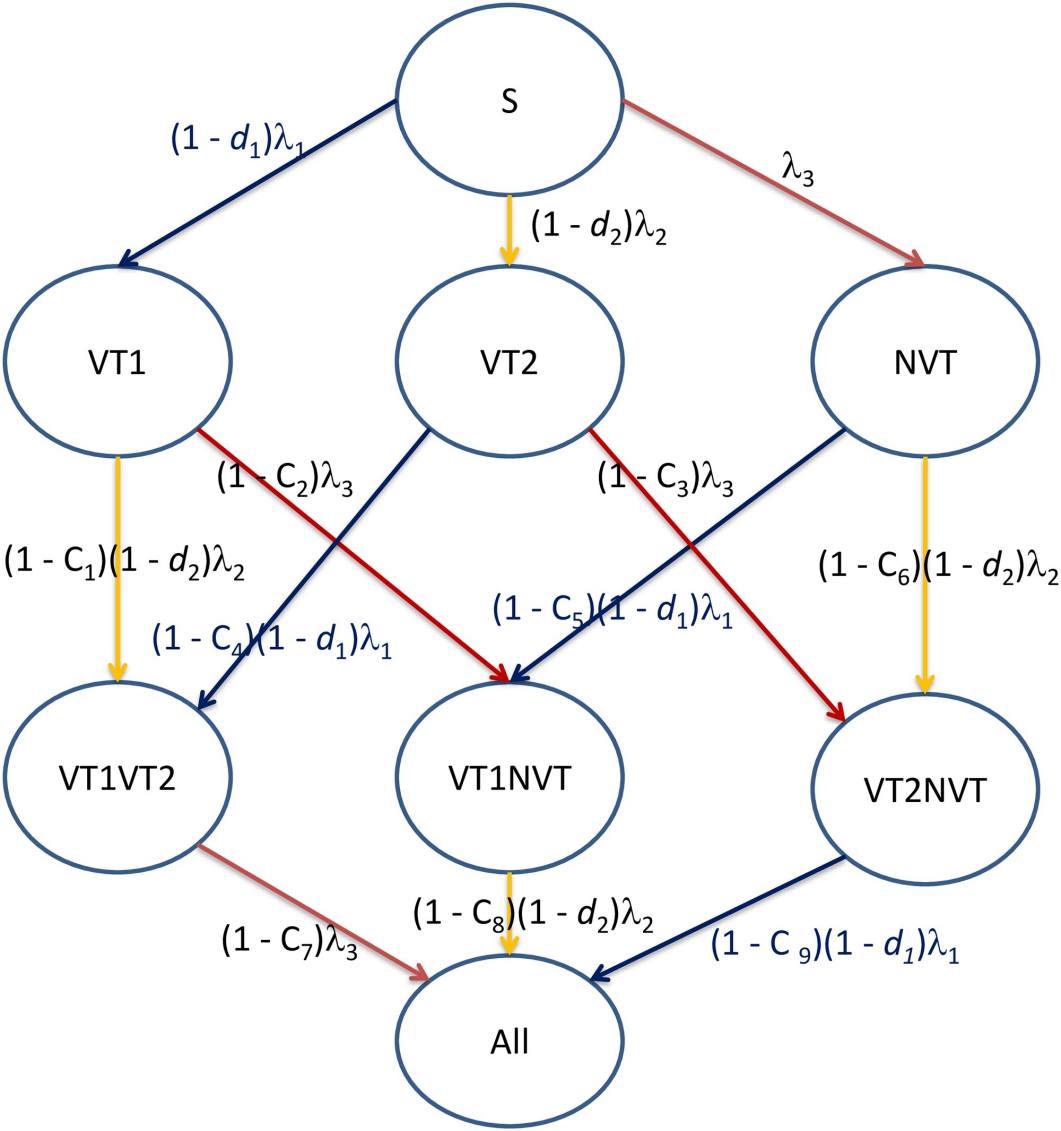
Flow diagram showing transitions between model compartments according to vaccine protection (different values of d1 and d2 as described in Fig 3). C variables, competition parameters; d1, vaccine efficacy against VT1 carriage; d2, vaccine efficacy against VT2 carriage; NVT, non-vaccine serotype group (non-PCV13 serotypes and serotype 3); PCV7, 7-valent pneumococcal conjugate vaccine; PCV13, 13-valent pneumococcal conjugate vaccine; S, susceptible; VEc, vaccine efficacy against carriage; VT1, vaccine serotype group 1 (PCV7 serotypes); VT2, vaccine serotype group 2 (serotypes only in PCV13 not in PCV7 excluding 1 and 3).

The population in the model is divided into 100 annual age cohorts (0, 1, 2, 3, …, 99). Each annual age cohort is divided into 48 equal-sized age cohorts (in total, 4,800 age cohorts in the total population in the model). In the absence of vaccination, individuals are born susceptible (S) to pneumococcal carriage and become infected with a VT1, VT2, or NVT serotype, as determined by the serogroup-specific force of infection (FOI), denoted as *λ*1, λ2, and λ3, respectively, in Fig 2. An episode of carriage does not result in protection against subsequent carriage of any serotype (i.e., susceptible-infectious-susceptible [SIS] model structure). If invasive disease occurs as a result of carriage, it is assumed to do so at the time of carriage acquisition, whereas transmission can occur at any time during the carriage episode. Individuals clear their infection with age-dependent clearance rates as estimated previously [4] and become susceptible again, but if concomitantly infected with a serotype from another grouping they move to a coinfected compartment (VT1VT2, VT1NVT, or VT2NVT). Individuals can potentially carry serotypes from all three serotype groupings, denoted as the ‘All’ compartment in Fig 2. Individuals already carrying a serotype from one of the three groupings will have some degree of protection against infections from another serotype grouping, according to the level of competition (C) between the three serotype groupings (parameters C_1_–C_9_ in Fig 2 (implemented as a reduced FOI). If no such competition exists (C = 0 and 1 –C = 1), the PCV programme will have the maximum reduction in overall IPD cases without any serotype replacement. With the other extreme scenario, where C = 1, the parameter 1 –C becomes zero, preventing coinfection, and maximum serotype replacement will occur after vaccination.

**Fig 3:**
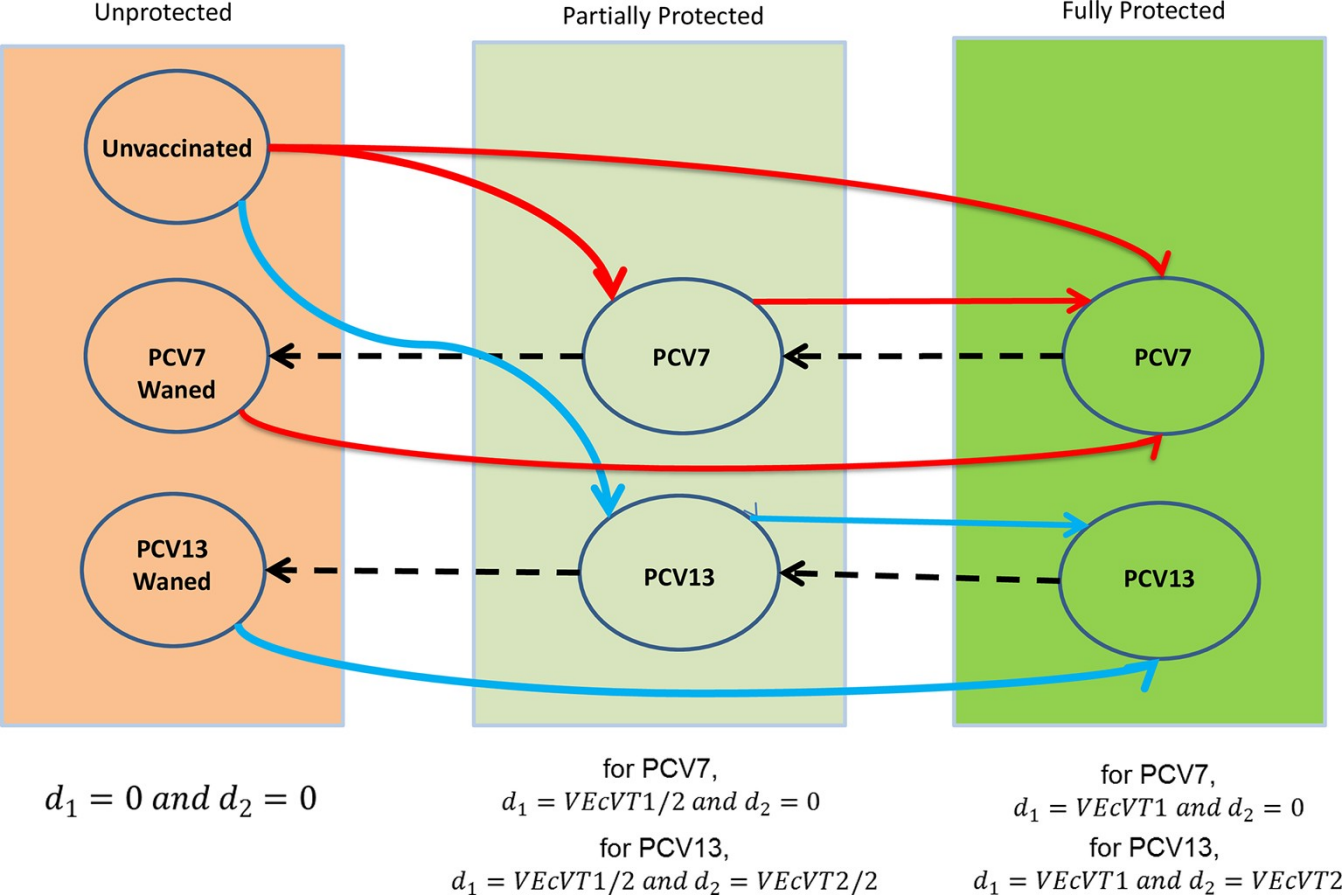
Vaccine protection status changes according to vaccine coverage (solid lines) and waning (dashed lines) with PCV7 and PCV13 (red arrows for PCV7 and blue arrows for PCV13 vaccination). The transition from unvaccinated to full protection after a single dose of PCV7 (top red line) represents children aged 1 to <2 years who received a single dose in the 2006 catch-up. PCV7, 7-valent pneumococcal conjugate vaccine; PCV13, 13-valent pneumococcal conjugate vaccine; VEc, vaccine efficacy against carriage; VT1, vaccine serotype group 1 (PCV7 serotypes); VT2, vaccine serotype group 2 (serotypes only in PCV13 not in PCV7 excluding 1 and 3).

The competition parameters C_1_ and C_2_ mainly drive the serotype replacement after PCV7 and C_3_ after PCV13 introduction, as the other six competition parameters become insensitive once VT1- and VT2-type carriage decreases postvaccination [9]. As in the earlier PCV13 model [9], these insensitive competition parameters were set at 0.5. C_1_ and C_2_ can be estimated from the replacement effect on VT2 and NVT after PCV7 introduction, while C_3_ influences the replacement of VT2 with NVT after PCV13 introduction. Detailed equations for Figs 2 and 3 are presented in S1 and S2 Equations.

### Vaccine efficacy parameters

Within the vaccine protected group, two doses in the first year of life or one dose after 12 months of age is assumed to confer the maximum degree of protection that can be obtained from PCV13 against carriage acquisition of the respective serotype grouping (VEcVT1 and VEcVT2 in Fig 3), here termed ‘full protection’. A single dose in the first year of life is assumed to provide half the maximum protection (VEcVT1/2) or VT2 (VEcVT2/2), here termed ‘partial protection’ [20,21]. Full protection wanes exponentially, with individuals then becoming partially protected; partial protection also wanes exponentially and individuals then move back to the fully susceptible group in Fig 3. The degree of protection and the waning function for the VT1 and VT2 groups are inversely correlated and cannot be estimated independently. For the base case, the average duration of protection for both full and partial protection was therefore set at 5 years, as in the previous PCV13 modelling study [9].

Vaccine efficacy against IPD (VEd) given carriage of a VT1 or VT2 serotype for those with two priming doses and/or a single dose in the second year of life, and those in receipt of a single dose under a year, is assumed to be 100% at the time of vaccination and to wane with the same average duration as for the full and partial protection, respectively, against carriage. S1 Fig illustrates how overall protection against carriage acquisition and development of IPD (given carriage) and carriage would wane with time since last dose, with an average duration of protection of 5 years and vaccine efficacy against carriage (VEc) for full protection of 60% as an example. With these assumed values, VEd post-booster reduces to 20% and VEc to <20% after 9 years.

The propensity to develop invasive disease upon carriage acquisition within each age group will be determined by the overall case-carrier ratio (CCR) of serotypes comprising the VT1, VT2, and NVT groupings and is derived from the ratio of IPD cases: incident carriage infections within each serotype grouping in each age group.

### Model selection

The model consisted of a static pre-vaccination equilibrium component and postvaccination dynamic component. Models of progressively increasing complexity were fitted to IPD data from the pre-PCV7 baseline and post-PCV period. In each model investigated, there were two parameters for the degree of vaccine protection against colonisation for VT1 and VT2, with, in the simplest model, three competition parameters that did not vary by age. Using the simplest model, three scenarios were investigated as potential causes for the rapid increase in NVT IPD cases from 2014/2015. The large rise in non-PCV13 IPD occurred shortly after the introduction of live attenuated influenza vaccine (LAIV) in a phased national programme starting with the youngest children in the 2013/2014 winter season and with older cohorts added sequentially in successive years. There is evidence that LAIV can increase carriage density in those already carrying *S*. *pneumoniae*, which might enhance transmission [22]. The model was therefore used to investigate the potential impact of a transient increase (ranging between 0% and 100%) in the FOI due to the LAIV programme, assuming that vaccinated children had an increased FOI for a month in November in each year (LAIV scenario). The PHE LAIV age-specific coverage data used are presented in S2 Fig. A carriage study conducted in England in 2015/2016 showed that while no individual non-PCV13 serotype showed an increase in invasiveness since 2012/2013, due to the changing mix of non-PCV13 serotypes in the nasopharynx, the CCR of this group as a whole had increased by around 50% [23]. There was also a small nonsignificant increase in the prevalence of non-PVC13 serotypes in carriage in 2015/2016 compared with 2012/2013, from 23.6% to 27%, possibly indicating a potential increase in the FOI of non-PCV13 serotypes. For the second and third scenarios, we therefore considered a one-time proportional increase between 0% and 100% in FOI or CCR in NVT, respectively, from 2014/2015 from the values estimated from the pre-PCV era (FOI or CCR scenarios). The scenario that best reproduced the observed trends in IPD up to 2015/2016 was then used in the model to investigate the impact of a change to a 1+1 PCV13 schedule from September 2018.

Using a Poisson likelihood to link the IPD cases with the output of the model, we ran a Nelder–Mead method to search for the maximum-likelihood estimate [24]. This was used to generate an Akaike Information Criterion (AIC) score for each of the scenarios. The CCR scenario had the lowest AIC value (see S1 Table), and this scenario was then used for exploring more complex models in which the competition parameters could vary by age group, with the most complex model having six age groups. In the latter model, the proportional increase in CCR was also allowed to vary across the six age groups (see S2 Table). Again, Poisson likelihood was used to select the best fitting model. These different levels of complexity were investigated in order to accommodate differences between age groups in the combination of serotypes comprising the NVT group [12].

### Model fitting

Table 1 summarises how the parameters in the model were derived and those that were varied in the fitting and sensitivity analysis. To find the best fitted model, parameter sets were drawn using independent uniform (between 0 and 1) distributions to find the set of model parameters with the maximum likelihood, using the Nelder–Mead method [24]. The estimation of the set of parameters for the pre-vaccination static model was performed by running it jointly with the postvaccination dynamic model (see the details and the flowchart of the overall model fitting procedures presented in S3 Fig).

**Table 1:** Summary of assumed, calculated, and the 26 fitted model parameter used in the final model and those that were varied in the fitting and sensitivity analysis.

Parameter	How derived	Comment
Duration of a carriage episode in days by age: 72 in 0–2-year-olds, 28 in 2–4-year-olds, 18 in 5–17-year-olds, 17 in 18+-year-olds	Fitting a Markov chain model to household transmission data in pre-PCV7 longitudinal carriage study	As derived in [4] and assumed to be the same for VT1, VT2, and NVT groups as shown in Cauchemez and colleagues [25]
Incidence of new carriage episodes by VT1, VT2, or NVT serogroup	Fitting a pneumococcal static model to pre-PCV7 longitudinal carriage study [13]	By three serogroups and seven age groups (see S3 Table for fitting results)
Duration of vaccine protection against carriage by VT1 and VT2 groups	Assumption: full protection wanes to partial protection, with an average duration assumed to be 5 years, and another 5 years for the average duration of partial protection to wane to zero	In the model fitting, duration is inversely related to degree of protection and so cannot be estimated independently. Duration was therefore fixed at 5 years [5]. In the sensitivity analysis, durations of 3 and 8 years were evaluated
Degree of full vaccine protection against carriage of VT1 and VT2 (two fitted parameters, VEcVT1 and VEcVT2) estimated at 0.55 (0.53, 0.57) and 0.30 (0.26, 0.36), respectively	Fitting a dynamic transmission model to changes in the pre- and post-PCV7 and post-PCV13 IPD data	For those whose immunity has waned to the partial protection category or who had only received one dose under a year, the degree of protection was assumed to be half that of the full protection. The scenario of a ‘0’+1 schedule was implemented to assess the effect of reducing the partial protection of one priming dose to zero
Competition parameters: C1, C2, C3 in six age groups (18 fitted parameters, see Table 2)	Fitting a dynamic transmission model to changes in the pre- and post-PCV7 and post-PCV13 IPD data	The magnitude of the increase in NVT IPD cases is determined by these competition parameters. The higher the level of competition, the greater the increase in NVT IPD cases. In the fitting, competition parameters were assumed to be age independent or allowed to vary between age groups (*n* = 2, 3, or 6)
Duration of vaccine protection against IPD	Assumption	An exponential decay function with the same waning parameter as used for VEc. Duration varied for the sensitivity analysis to 3 and 8 years
Degree of vaccine protection against IPD (VEd) given carriage acquisition	Fixed at 100% while fully or partially protected	The fitting resulted in about 100% before any waning, so this parameter was fixed at 100%. Despite seeming high, given waning, the overall protection against IPD as a function of time since vaccination appears realistic (see S1 Fig)
Proportional increase in CCR of NVTs from 2014/2015 in six age groups (6 fitted parameters, see Table 2)	Fitting a dynamic transmission model to changes in the post-PCV13 IPD data from 2014/2015 to 2015/2016	One-time proportionate increase is assumed to continue out to 2030/2031
Transmission probabilities per contact, FOI, CCR by age groups and serogroups	Calculated from the pre-vaccination static model using the chosen competition parameters and the prevalence data	Given the 18 fitted competition parameters in the final model, one set of the transmission probabilities per contact, FOIs, and CCRs could be obtained by directly solving the static model equations using the observed pre-PCV carriage prevalence by age groups and serotype groups and the Nelder–Mead method [24]

Abbreviations: CCR, case-carrier ratio; FOI, force of infection; IPD, invasive pneumococcal disease; NVT, non-vaccine serotype group (non-PCV13 serotypes and serotype 3); PCV, pneumococcal conjugate vaccine; PCV7, 7-valent pneumococcal conjugate vaccine; PCV13, 13-valent pneumococcal conjugate vaccine; VEc, vaccine efficacy against carriage; VEd, vaccine efficacy against IPD; VT1, vaccine serotype group 1 (PCV7 serotypes); VT2, vaccine serotype group 2 (serotypes only in PCV13 not in PCV7 excluding 1 and 3).

For the pre-vaccination equilibrium fitting, the FOIs by the seven age groups (under 1, 1–2, 3–4, 5–9, 10–19, 20–39, and 40+ years) were obtained for the given set of competition parameters by fitting the static model to the pre-PCV7 carriage prevalence data. Details of the fitting process are given in S1 Text. Using these calculated FOIs, the transmission probabilities per contact in seven age groups were calculated using the 2005 population data and the POLYMOD close contact matrix [16], supplemented with the additional data on under-1-year-olds [17]. The numbers of new infection estimates were used as denominators to estimate CCRs by 16 age groups (<2 months, 2–3 months, 4–5 months, 6–11 months, 1 year, 2 years, 3 years, 4 years, 5–9 years, 10–14 years, 15–24 years, 25–44 years, 45–64 years, 65–74 years, 75–84 years, and 85+ years) and three serotype groupings using the pre-PCV7 average IPD cases as numerators. Where the age group for the carriage data was coarser than that for IPD incidence, the average carriage prevalence in the coarser age group that encompassed the finer IPD age groups was used. The finely stratified age groups under one year for estimating CCRs were chosen to reflect the vaccination effect by numbers of primary doses [5].

With the calculated transmission probabilities per contact and CCR by age groups and serogroups from the pre-vaccination fitting, the postvaccination dynamic model ran for 11 years between 2005/2006 and 2015/2016 to fit to post-PCV IPD data.

### Selection of parameter sets for long-term simulation

For assessing the future impact of schedule changes, we used the model with age-dependent competition parameters and age-dependent NVT increases (a total of 26 age-dependent fitted parameters), as this had the lowest AIC value (see S1 Text). We then identified 500 parameter sets for which each of the 26 parameter values was within ±0.3 of its maximum likelihood value and for which the overall likelihood of the parameter was close to the maximum likelihood. The range of ±0.3 was sufficiently wide that the selected parameter sets did not give values close to the ends of these ranges for most parameters (Table 2). Results are shown as the median value of the accepted 500 parameters sets and the minimum to maximum range is shown as the uncertainty interval (UI).

**Table 2:** The medians of 500 parameter sets (UI) of the estimated competition parameters by age group (units indicate the degree of competition between 1 and 0, 0 = no competition and no serotype replacement) and proportional increase in CCR of the NVT group from 2014/2015 by comparing the model outputs to the IPD cases by three pneumococcal serotype groups and age groups in England and Wales between 2005/2006 and 2015/2016; base case scenario with 5 years average duration of protection.

Age group (years)	<2	2–4	5–14	15–44	45–64	65+
C1	0.74 (0.38, 0.93)	0.01 (0.00, 0.46)	0.00 (0.00, 0.30)	0.26 (0.04, 0.63)	0.43 (0.16, 0.75)	0.51 (0.19, 0.79)
C2	0.08 (0.00, 0.38)	0.00 (0.00, 0.30)	0.00 (0.00, 0.29)	0.00 (0.00, 0.32)	0.13 (0.00, 0.49)	1.00 (0.70, 1.00)
C3	0.84 (0.63, 1.00)	0.07 (0.00, 0.49)	0.36 (0.10, 0.70)	0.50 (0.18, 0.77)	0.72 (0.42, 1.00)	0.86 (0.56, 1.00)
CCR increase (proportion)	0.41 (0.09, 0.68)	0.36 (0.00, 0.56)	0.00 (0.00, 0.29)	0.00 (0.00, 0.14)	0.10 (0.00, 0.33)	0.67 (0.43, 0.94)

Abbreviations: CCR, case-carrier ratio; IPD, invasive pneumococcal disease; NVT, non-vaccine serotype group (non-PCV13 serotypes and serotype 3); UI, uncertainty interval.

### Long-term simulations

Using the 500 sampled parameter sets, the model was used to simulate the future impact of a change from a 2+1 to a 1+1 schedule starting in September 2018. As a worst-case scenario, we modelled a ‘0’+1 schedule assuming that the first dose of a 1+1 schedule in infants whose mothers received a diphtheria-containing acellular pertussis vaccine in pregnancy provided no protection against carriage of vaccine-type serotypes or VT IPD due to impairment of responses to the cross-reacting material (CRM)-conjugated serotypes in PCV13, although the efficacy of the booster dose was unimpaired [7]. The impact of stopping PCV13 vaccination altogether in September 2018 was also evaluated.

### Sensitivity analysis

For exploring the effect of varying the waning function, we refitted the model using an average duration of protection for VEc and VEd of 3 and 8 years instead of 5 years as in the base case and compared the 2+1 schedule with the 1+1 schedule with the refitted parameters. We also refitted the model after excluding serotype 3 from the model and compared the 2+1 and the 1+1 schedule with the refitted parameters.

### Estimating the impact on pneumococcal-attributable non-bacteraemic pneumonia

The relationship between carried serotypes and those causing non-bacteraemic pneumococcal pneumonia, and the effect of PCV vaccination on this relationship, is not as well documented as that for IPD. It was therefore assumed that the percentage difference in cumulative IPD cases over the first 5 years between a 2+1 and a 1+1 schedule (base case assumptions) would also apply to age-specific incidence estimates of pneumococcal-attributable community-acquired pneumonia (CAP). For adults aged ≥16 years, the incidence of pneumococcal-attributable CAP post-PCV was derived from a study of admissions for CAP to a hospital in Nottingham, England, from 2008/2009 to 2015/2016, in which cases were defined as CAP based on clinical and radiological criteria and a pneumococcal aetiology was confirmed by a urinary pneumococcal antigen detection method supplemented by a serotype-specific urinary assay ([26] and personal communication from Dr. Wei Shen Lim, Nottingham University Hospitals NHS Trust). The proportion of CAP in adults that was identified as pneumococcal attributable reduced over the study period from 40% in 2008 to 30% in 2015/2016. This latter proportion was assumed to have continued to September 2018, when the 1+1 schedule was implemented in the model. In the pneumococcal CAP sensitivity analysis, we assumed that half the true cases were missed in the Nottingham studies due to lack of sensitivity of the assay and failure to include all potentially eligible cases. For children <16 years of age, comparable data on pneumococcal-attributable CAP post-PCV do not exist. Instead, it was conservatively assumed that 50% of hospital admissions for pneumonia of unspecified cause (ICD-10 diagnosis code J18) before the introduction of PCV were pneumococcal attributable [27]. By March 2015, there was a 30% reduction in J18 in children <16 years old [28], which, if due to a specific effect of the PCV programme, would mean that only 28% of J18 admissions in children by March 2015 were pneumococcal attributable. This percentage was assumed to continue until September 2018. The percentage change in IPD predicted as a result of the schedule change was therefore applied to 28% of the J18 incidence from the Hospital Episode Statistics at the time of the schedule change [28].

### Impact on pneumococcal-attributable deaths

The case fatality rate (CFR) by age for IPD was obtained from studies of laboratory confirmed cases in England and Wales and Canada [29–31]. The CFR for pneumococcal CAP in those under 45 years is less than 1% and was derived from a study in England using hospital admissions from 1995 to 2000 [32]. The CFRs for CAP are higher in adults and have declined in recent years [33], and were obtained from a study in 17 countries that included data to 2011 [34]. The age-specific CFRs used in the study are shown in S4 Table.

## Results

### Pre- and post-PCV fitting

**Fig 4:**
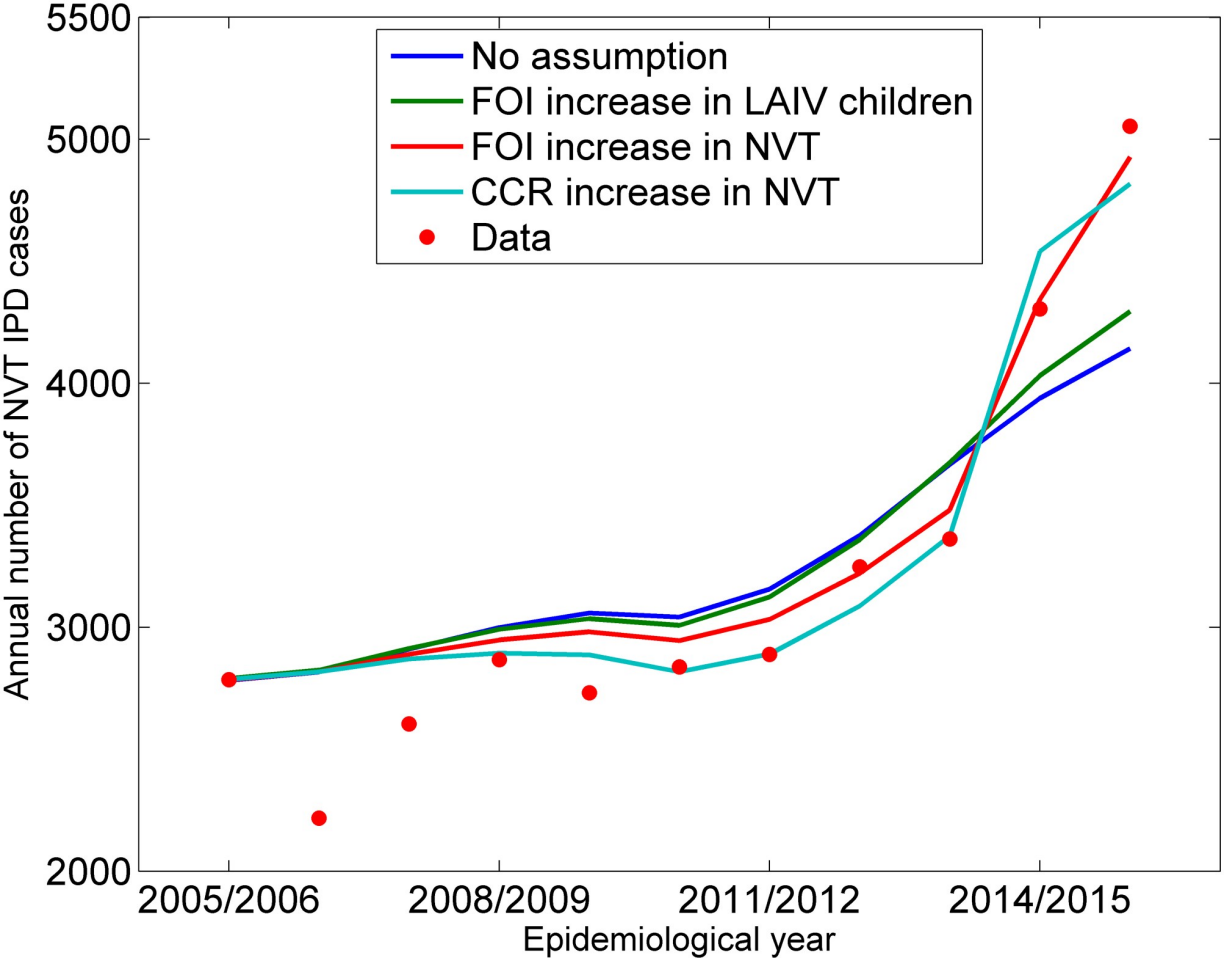
NVT IPD cases: Best fitting model prediction under the base case, LAIV, NVT FOI, and NVT CCR increase scenarios compared with NVT data for all ages. Base case scenario, FOI and CCR unchanged from the pre-PCV7 era (no assumption scenario); LAIV scenario, a temporary FOI increase among LAIV vaccinated children; NVT FOI increase scenario, FOI increase in NVT from 2014/2015 (age independent); NVT CCR increase scenario, CCR increase in NVT from 2014/2015 (age independent). CCR, case-carrier ratio; FOI, force of infection; IPD, invasive pneumococcal disease; LAIV, live attenuated influenza vaccine; NVT, non-vaccine serotype group (non-PCV13 serotypes and serotype 3); PCV7, 7-valent pneumococcal conjugate vaccine; PCV13, 13-valent pneumococcal conjugate vaccine.

As expected, the scenario with the FOI and CCR parameters for the NVT group derived from the pre-PCV7 era with no assumption about the potential cause of the rapid increase in NVT from 2014/2015 did not fit well the post-PCV IPD data from 2014/2015 onwards (Fig 4). Similarly, the LAIV scenario did not produce a good fit, even with a 100% increase in the FOI for one month for those children who received LAIV. Better fits were produced by scenarios in which there was an age-independent proportional increase in the FOI or CCR of the NVT group overall, with the best fit for the latter (i.e., the lowest value for the AIC) (S1 Table).

The model with the proportional increase in CCR of the NVT group from 2014/2015 was therefore used in the simulations to 2030/2031, with the assumption that the increase would persist. When investigating the effect of allowing the competition parameters and the proportionate increase in NVT CCR from 2014/2015 to vary by age, the best fit (lowest AIC value) was obtained with six age groups for both the competition parameters and the NVT CCR increase (See S2 Table). The range of parameter values in the 500 accepted parameter sets are shown in Table 2.

**Fig 5:**
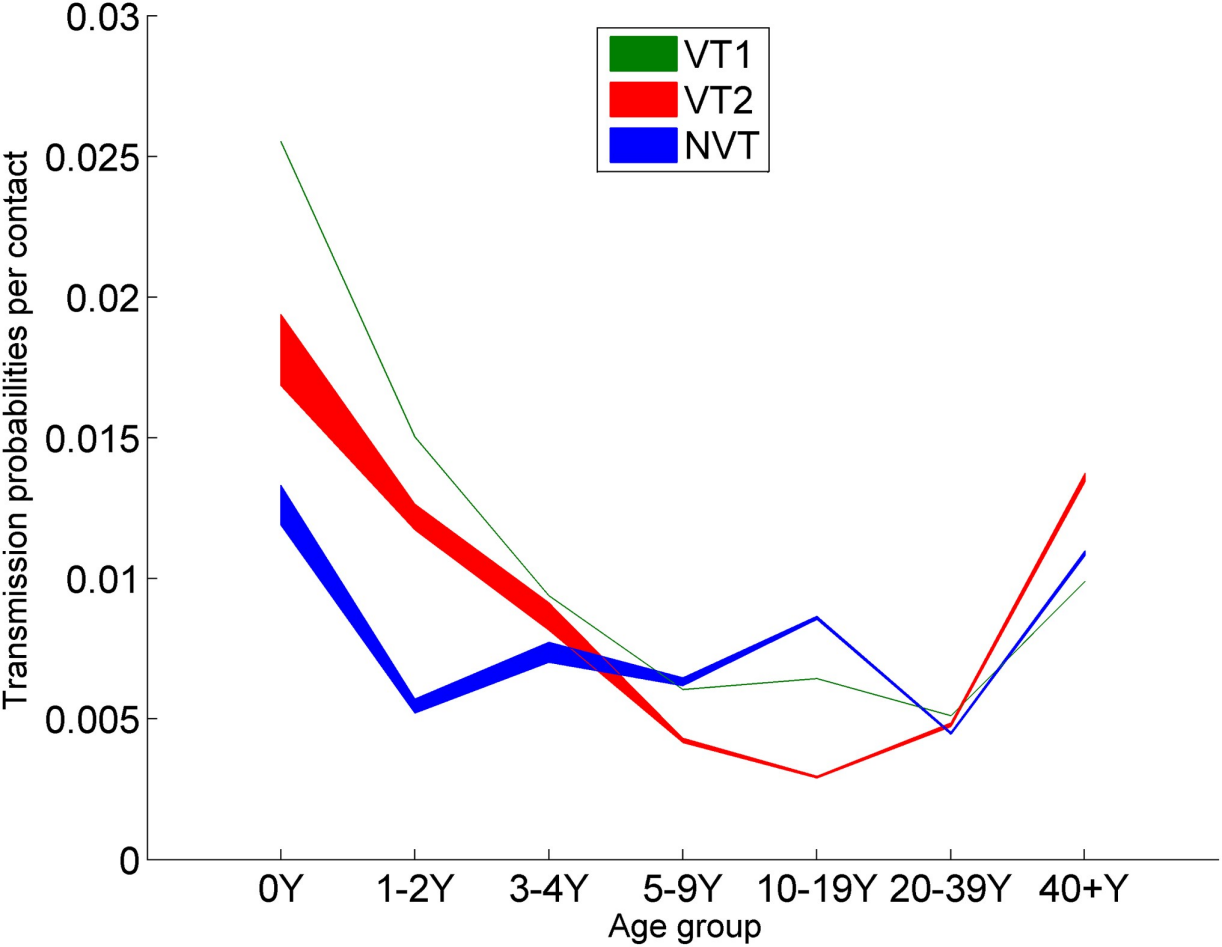
Transmission probabilities per contact by seven age groups (0 year, 1–2 years, 3–4 years, 5–9 years, 10–19 years, 20–39 years, and 40+ years) and three serogroups (VT1, VT2, and NVT), obtained by fitting the longitudinal carriage swab data in 2001/2002 [13]. UI is the range of the 500 accepted model parameter sets. NVT, non-vaccine serotype group (non-PCV13 serotypes and serotype 3); PCV7, 7-valent pneumococcal conjugate vaccine; PCV13, 13-valent pneumococcal conjugate vaccine; UI, uncertainty interval; VT1, vaccine serotype group 1 (PCV7 serotypes); VT2, vaccine serotype group 2 (serotypes only in PCV13 not in PCV7 excluding 1 and 3).

**Fig 6:**
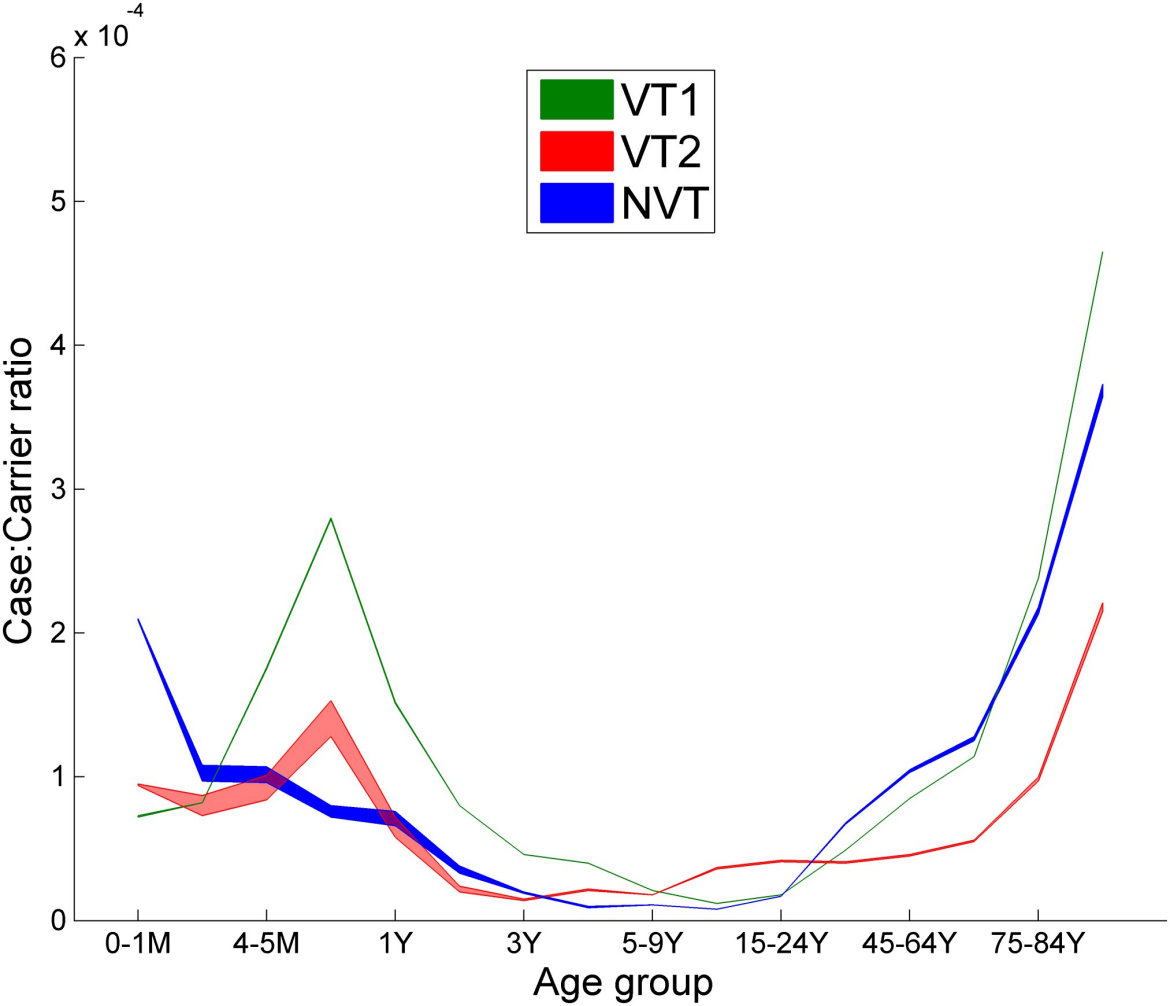
CCRs for 16 age groups (0–1 months, 2–3 months, 4–5 months, 6–11 months, 1 year, 2 years, 3 years, 4 years, 5–9 years, 10–14 years, 15–24 years, 25–44 years, 45–64 years, 65–74 years, 75–84 years, and 85+ years) and three serogroups (VT1, VT2, and NVT), obtained by fitting the longitudinal carriage swab data in 2001/2002 [13] and 6-year average IPD cases in England and Wales between 2001/2002 and 2005/2006. UI is the range of the 500 accepted model parameter sets. CCR, case-carrier ratio; IPD, invasive pneumococcal disease; NVT, non-vaccine serotype group (non-PCV13 serotypes and serotype 3); PCV7, 7-valent pneumococcal conjugate vaccine; PCV13, 13-valent pneumococcal conjugate vaccine; UI, uncertainty interval; VT1, vaccine serotype group 1 (PCV7 serotypes); VT2, vaccine serotype group 2 (serotypes only in PCV13 not in PCV7 excluding 1 and 3).

VEcVT1 and VEcVT2 were estimated as 0.55 (UI 0.53, 0.57) and 0.30 (UI 0.26, 0.36), respectively, implying that PCV7 is more efficacious against carriage acquisition of VT1 serotypes than PCV13 against carriage acquisition of the four additional serotypes in the VT2 group (5, 7F, 6A, and 19A). The 65+ age group requires the largest proportional increase of 67% in NVT CCR to describe the rapid increase of the NVT IPD cases since 2014/2015 [12]. Model results for the transmission probabilities per contact and the CCRs by age group under the base case are shown in Figs 5 and 6, respectively. The results indicated that in older age groups, the CCR of the NVT exceeded that of the VT2 group. In the sensitivity analysis, exclusion of serotype 3 from the model resulted in a lower CCR for the NVT group than in the base case, when it was included as an NVT, although in the older age groups the NVT CCR was still higher than for the VT2 group (data not shown).

**Fig 7:**
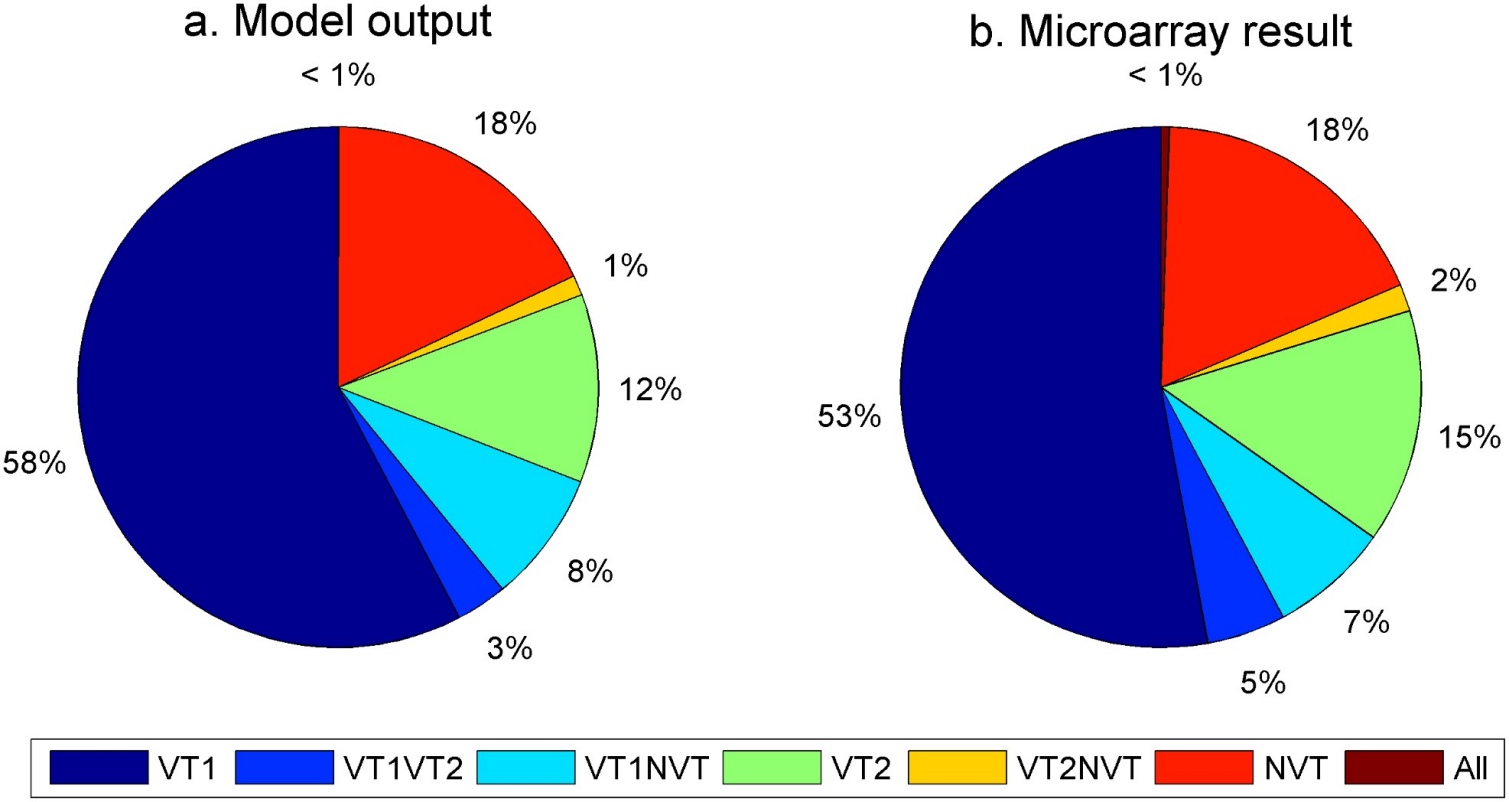
Pie charts of (A) model result from the fitting (using the maximum likelihood parameter set) and (B) the microarray-generated data [14] from the pre-PCV7 longitudinal study of the proportion of carriers in the different model compartments among those under 5 years of age. NVT, non-vaccine serotype group (non-PCV13 serotypes and serotype 3); PCV7, 7-valent pneumococcal conjugate vaccine; PCV13, 13-valent pneumococcal conjugate vaccine; VT1, vaccine serotype group 1 (PCV7 serotypes); VT2, vaccine serotype group 2 (serotypes only in PCV13 not in PCV7 excluding 1 and 3).

The proportion of children under 5 years of age who were carrying single or multiple serotypes in the pre-PCV7 period estimated from the fitting compared with the pre-PCV7 carriage data generated with the microarray method [14] showed a close correspondence (Fig 7a and 7b, respectively).

**Fig 8:**
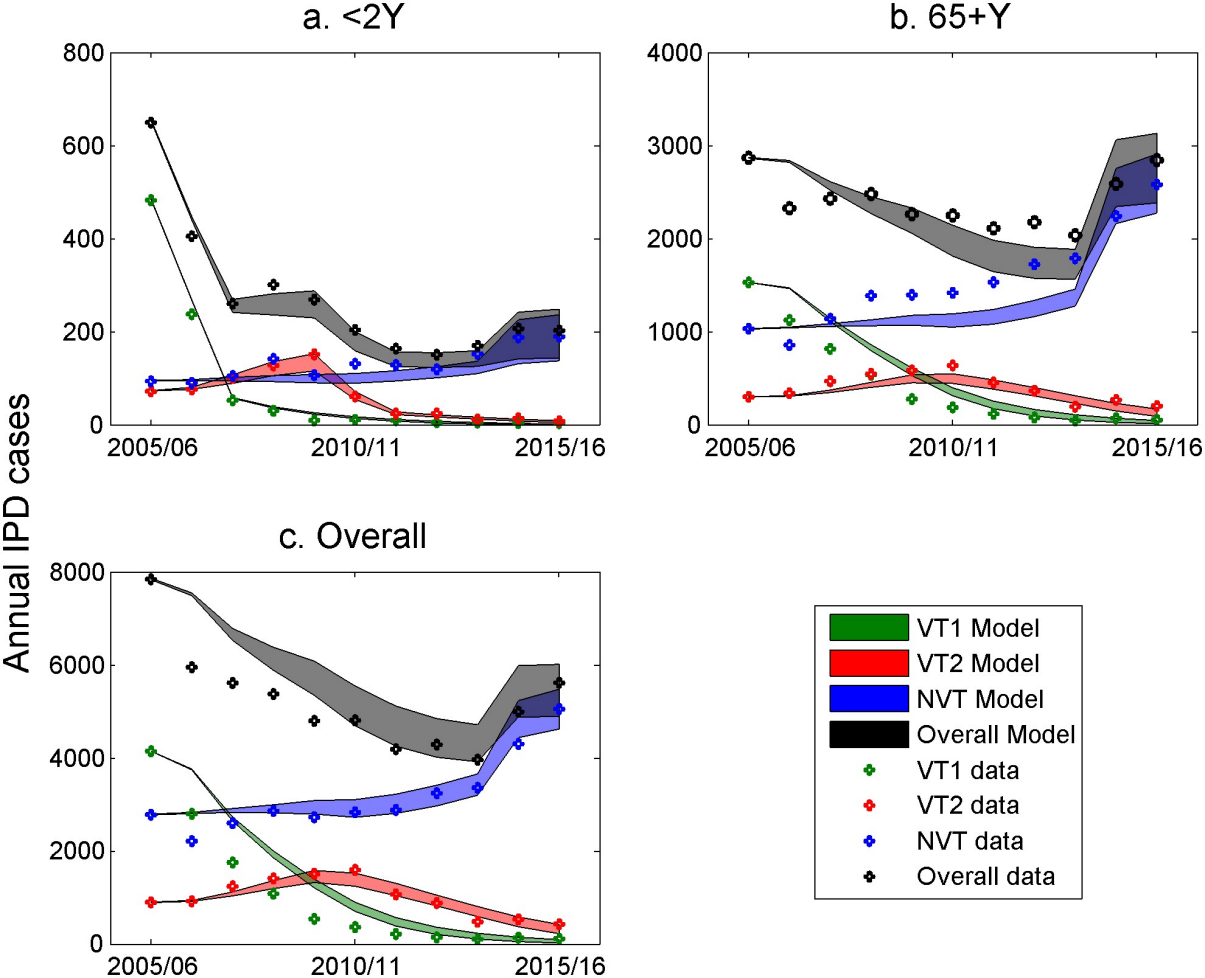
Comparison of the annual overall IPD data points (circles) and fitted model outputs with the 500 parameter sets between 2005/2006 and 2015/2016 in England and Wales; the width of the lines represents the UI. (a) Under-2-year-olds, (b) 65+-year-olds, and (c) overall cases. IPD, invasive pneumococcal disease; NVT, non-vaccine serotype group (non-PCV13 serotypes and serotype 3); PCV7, 7-valent pneumococcal conjugate vaccine; PCV13, 13-valent pneumococcal conjugate vaccine; UI, uncertainty interval; VT1, vaccine serotype group 1 (PCV7 serotypes); VT2, vaccine serotype group 2 (serotypes only in PCV13 not in PCV7 excluding 1 and 3).

The fitting comparison between IPD data and model outputs for children <2 years, 65+ year olds, and all ages in the post-PCV13 period up to 2014/2015 is shown in Fig 8, with other age groups available in S4 Fig. There was a good fit between the model output and IPD data for <2-year-olds, but with underestimation of NVT IPD cases in 5–64-year-olds and overestimation in 65+-year-olds in the post-PCV7 period.

### Long-term simulation of potential PCV13 programmes

**Fig 9:**
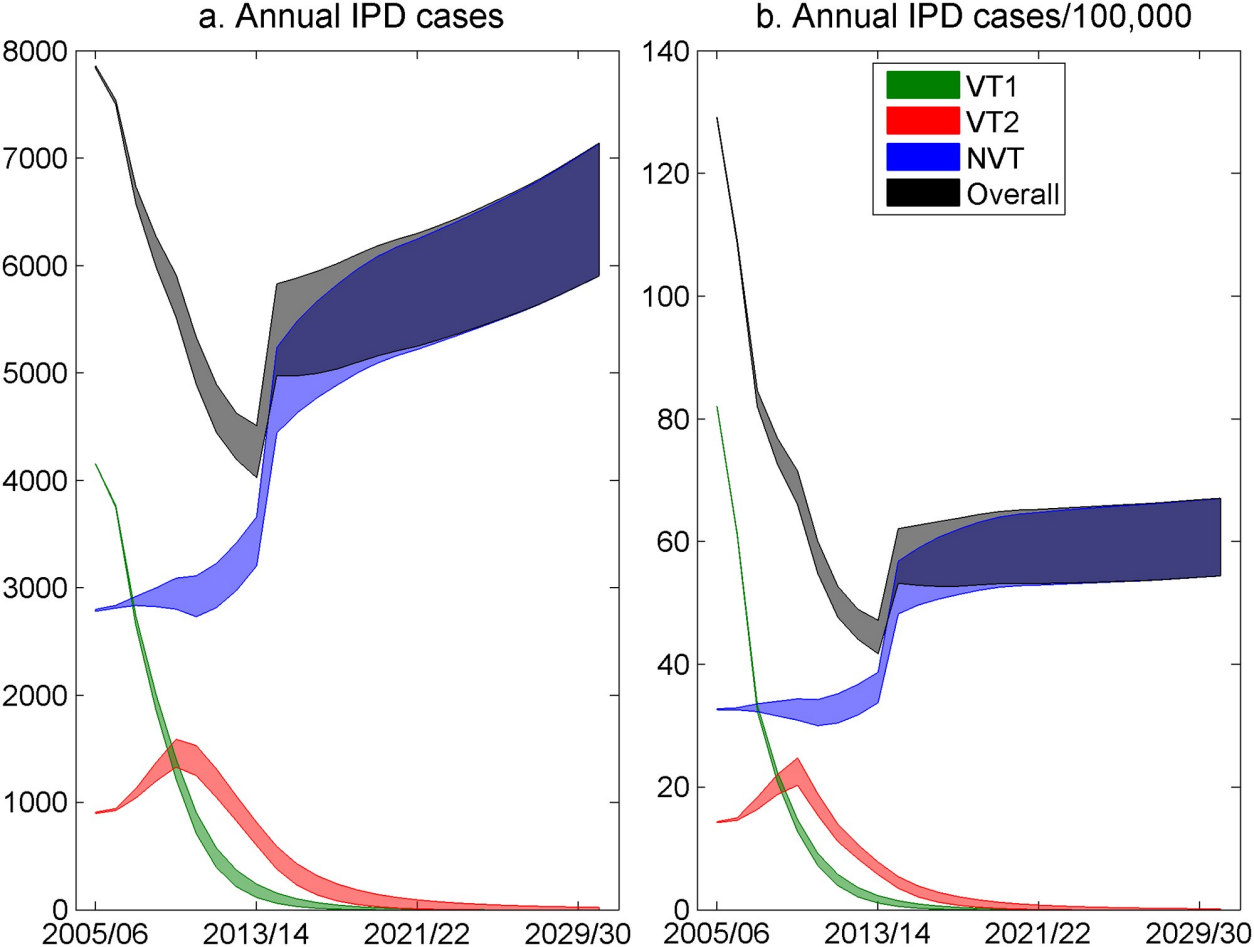
Simulated (a) annual IPD cases, all ages, and (b) annual IPD incidence/100,000 by serotype groupings between 2005/2006 (pre-PCV7 average) and 2030/2031 in England and Wales with the current 2+1 PCV13 schedule; the width of the lines represents the UI. IPD, invasive pneumococcal disease; NVT, non-vaccine serotype group (non-PCV13 serotypes and serotype 3); PCV7, 7-valent pneumococcal conjugate vaccine; PCV13, 13-valent pneumococcal conjugate vaccine; UI, uncertainty interval; VT1, vaccine serotype group 1 (PCV7 serotypes); VT2, vaccine serotype group 2 (serotypes only in PCV13 not in PCV7 excluding 1 and 3).

The simulated IPD cases and IPD incidence (all ages) by serotype grouping out to 2030/2031 are shown in Fig 9 in the base case with the 2+1 scenario. Due to the increase in the numbers of elderly individuals in the population in future years, overall IPD cases are predicted to increase, although incidence rates are predicted to level off around 2020 as the increase in NVT plateaus. In subsequent plots and tables, cases are shown and not incidence.

**Fig 10:**
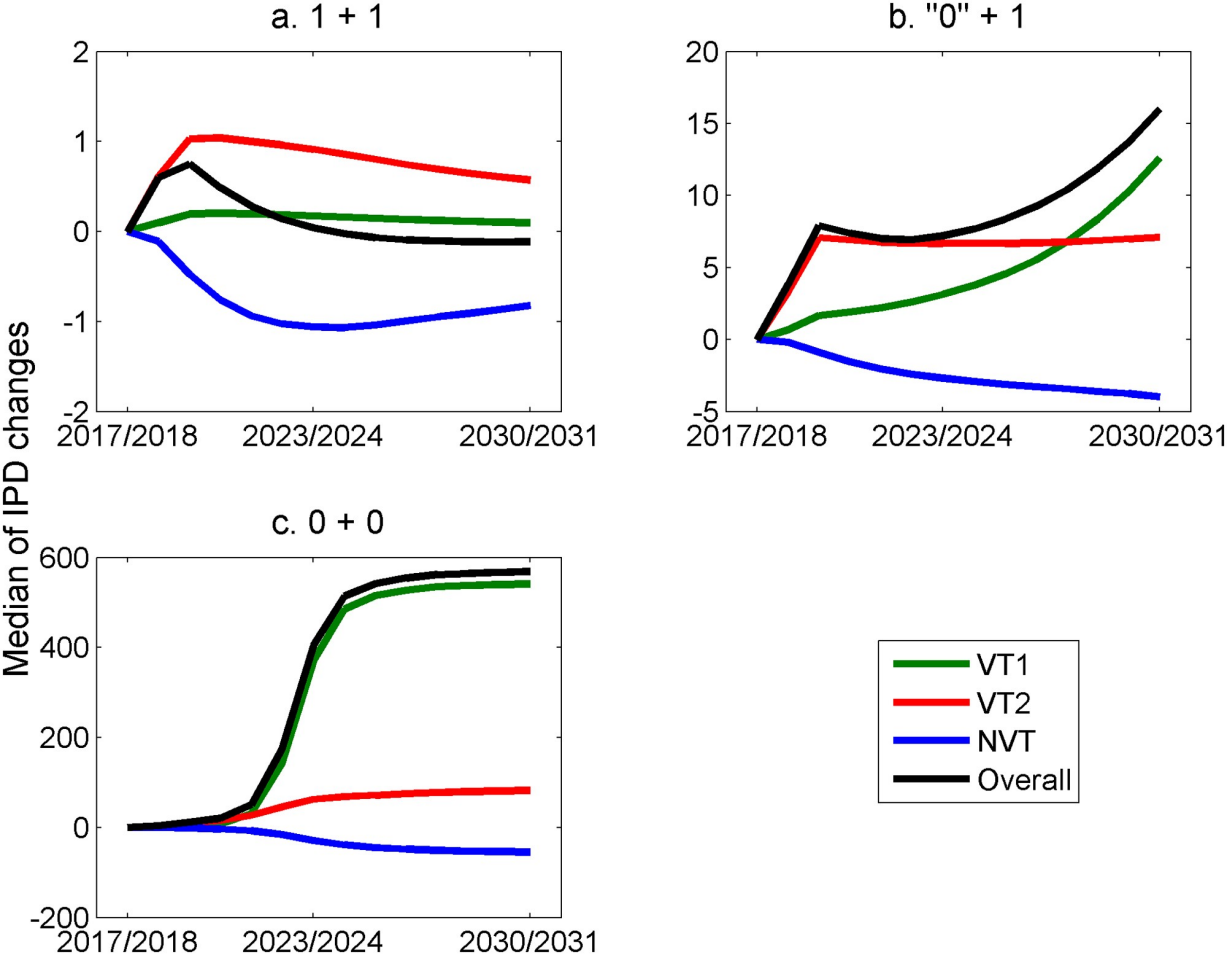
Predicted difference in the annual number of IPD cases, by serotype grouping and overall, in <2-year-olds if changing from September 2018 from the current 2+1 schedule to (a) a 1+1 schedule, (b) a ‘0’+1 schedule, and (c) a 0+0 schedule (stopping PCV13 vaccination). Median of long-term simulation results with the 500 accepted parameter sets. IPD, invasive pneumococcal disease; NVT, non-vaccine serotype group (non-PCV13 serotypes and serotype 3); PCV7, 7-valent pneumococcal conjugate vaccine; PCV13, 13-valent pneumococcal conjugate vaccine; VT1, vaccine serotype group 1 (PCV7 serotypes); VT2, vaccine serotype group 2 (serotypes only in PCV13 not in PCV7 excluding 1 and 3).

**Fig 11:**
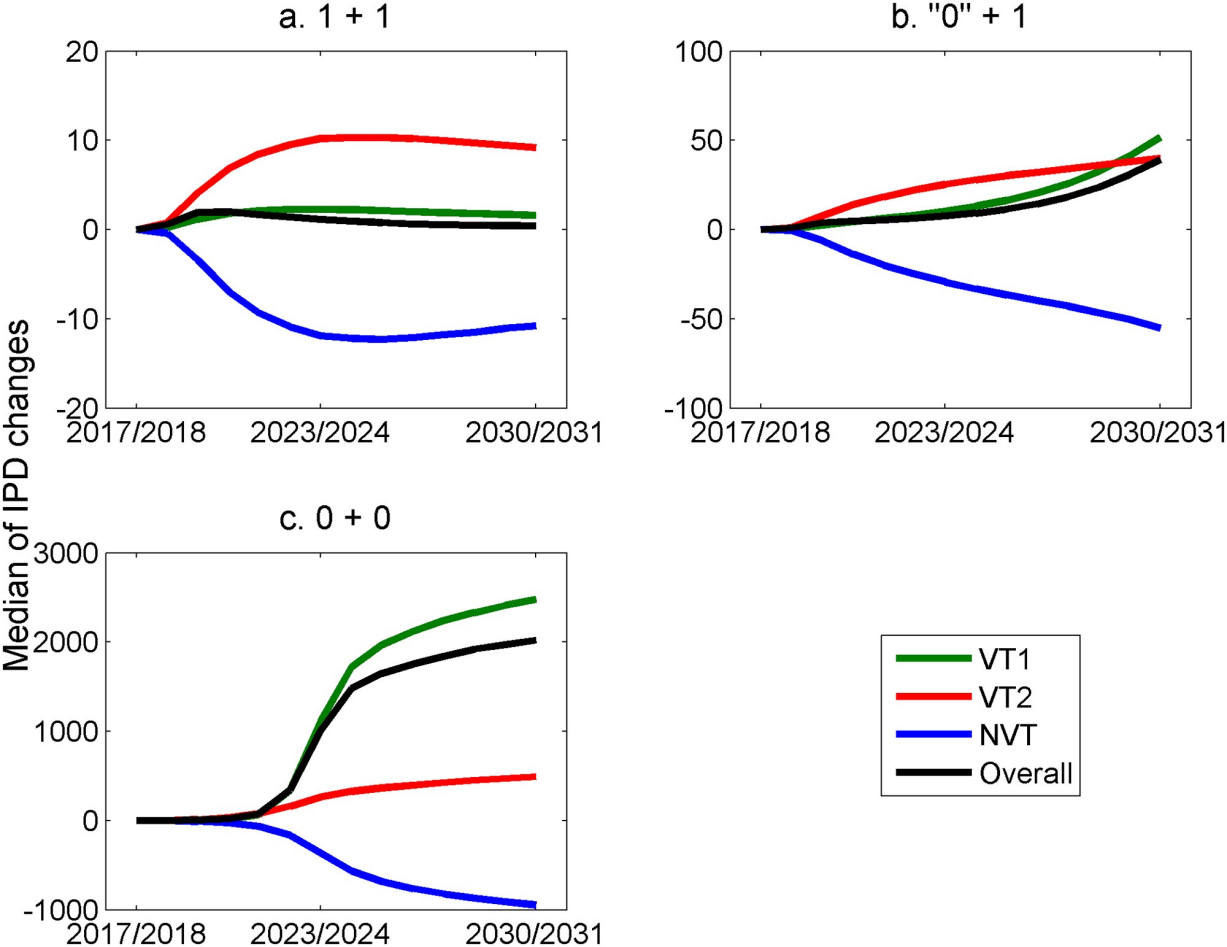
Predicted difference in the annual number of IPD cases, by serotype groupings and overall, in 65+-year-olds if changing from September 2018 from the current 2+1 schedule to (a) a 1+1 schedule, (b) a ‘0’+1 schedule, and (c) a 0+0 schedule (stopping PCV13 vaccination). Median of long-term simulation results with the 500 accepted parameter sets. IPD, invasive pneumococcal disease; NVT, non-vaccine serotype group (non-PCV13 serotypes and serotype 3); PCV7, 7-valent pneumococcal conjugate vaccine; PCV13, 13-valent pneumococcal conjugate vaccine; VT1, vaccine serotype group 1 (PCV7 serotypes); VT2, vaccine serotype group 2 (serotypes only in PCV13 not in PCV7 excluding 1 and 3).

The predicted changes in the annual numbers of IPD cases in under-2-year-olds and 65+-year-olds if changing from a 2+1 to a 1+1, ‘0’+1, or 0+0 (i.e., stopping PCV13 vaccination) schedule from September 2018 are shown in Figs 10 and 11, respectively.

The initial trends in children under 2 on changing to a 1+1 schedule (Fig 10a) reflect both the loss of direct protection in infants against VT1 and VT2 and the associated reduction in herd immunity (as seen in the accompanying increases in 65+-year-olds in Fig 11a). However, the model predicts that there will subsequently be a continued reduction in VT1 and VT2 cases as herd immunity is re-established. Under the ‘0’+1 schedule, with no protection against carriage or disease in infants under a year, the model predicts that after the initial sharp increase in under-2-year-olds, due to loss of direct protection, there will be a more gradual increase in VT1- and VT2-type cases due to a reducing herd immunity over time, as reflected in the trends in under-2-year-olds in Fig 10b and 65+-year-olds in Fig 11b. Under this extreme scenario, the number of additional cases is, however, relatively small. Stopping the PCV13 programme altogether (0+0) would result in a rapid return to pre-PCV7 levels within 5–6 years, with the annual number of IPD cases in under-2-year-olds approaching 600.

Table 3 gives the difference in the cumulative additional number of cases by age group during the first 5 years predicted to occur as a result of the change from the 2+1 to the three alternative schedules. S5 Fig shows the predicted total annual number of IPD cases by age group from 2018 out to 2030/2031 under the base case for the 1+1 schedule. The current benefit of the PCV13 programme, in terms of reduction in numbers of cases, compared with the pre-PCV7 period is predicted to be maintained in all age groups under 65 years of age.

**Table 3:** Median cumulative additional cases (UI) over the first 5 years (2018/2019 to 2023/2024) by a change from the 2+1 to three potential PCV13 schedules in England and Wales from September 2018.

Schedule	<2	2–4	5–14	15–44	45–64	65+	All
1+1	2 (0, 6)	0 (−1, 0)	1 (0, 2)	12 (5, 27)	9 (2, 21)	8 (−7, 25)	31 (6, 76)
‘0’+1	33 (15, 64)	0 (−1, 1)	2 (1, 5)	28 (12, 59)	22 (6, 49)	21 (−11, 66)	105 (35, 226)
0+0	264 (127, 454)	90 (39, 161)	23 (10, 41)	267 (126, 461)	289 (120, 537)	449 (148, 902)	1,374 (593, 2,535)

Abbreviation: PCV13, 13-valent pneumococcal conjugate vaccine; UI, uncertainty interval.

### CAP

Under the assumption that the change from a 2+1 to a 1+1 schedule would produce a similar percentage change in CAP as in IPD, the model predicted an additional 83 (−10, 242) CAP cases over the first 5 years under the base case scenario (Table 4). A sensitivity analysis with doubling the pneumococcal CAP cases from Rodrigo and colleagues [26] showed 149 (−11, 420) incremental pneumococcal CAP cases (Table 4). The impact on CAP cases under the ‘0’+1 schedule is shown in S5 Table.

**Table 4:** Percentage change in accumulated overall IPD cases (median, UI), incremental deaths due to IPDs, and potential incremental cases of, and deaths from, pneumococcal CAP for the first 5 years by age group when changing the current 2+1 schedule to 1+1 in England and Wales in September 2018. The sensitivity scenario presents the results when doubling the pneumococcal CAP incidence for those aged 15+ years from Rodrigo and colleagues [2,6].

Age (years)	Percentage change in IPD cases cumulative over 5 years	IPD deaths, cumulative over 5 years	Pneumococcal CAP					
			Base case			Sensitivity scenario		
			Annual CAP cases at time of the schedule change	Incremental CAP cases, cumulative over 5 years	Incremental CAP deaths, cumulative over 5 years	Annual CAP cases at the time of the schedule change	Incremental CAP cases, cumulative over 5 years	Incremental CAP deaths, cumulative over 5 years
<2	0.22% (0.03%, 0.74%)	0 (0, 0)	1,143	12 (1, 43)	0 (0, 0)	1,143	12 (1, 43)	0 (0, 0)
2–4	−0.04% (−0.20%, 0.06%)	0 (0, 0)	1,141	−2 (−12, 3)	0 (0, 0)	1,141	−2 (−12, 3)	0 (0, 0)
5–14	0.14% (0.03%, 0.36%)	0 (0, 0)	977	7 (2, 18)	0 (0, 0)	977	7 (2, 18)	0 (0, 0)
15–44	0.29% (0.11%, 0.65%)	1 (0, 3)	2,082	30 (11, 68)	1 (0, 2)	4,165	60 (23, 136)	2 (1, 4)
45–64	0.13% (0.03%, 0.31%)	1 (0, 3)	2,387	15 (4, 37)	1 (0, 2)	4,773	30 (7, 75)	1 (0, 3)
65+	0.05% (-0.04%, 0.17%)	2 (−2, 7)	8,442	21 (−16, 73)	2 (−2, 8)	16,883	42 (−33, 146)	4 (−4, 16)
All	0.11% (0.02%, 0.28%)	4 (−1, 12)	16,171	83 (−10, 242)	4 (−1, 12)	29,082	149 (−11, 420)	8 (−3, 23)

Abbreviations: CAP, community-acquired pneumonia; IPD, invasive pneumococcal disease; UI, uncertainty interval.

### Deaths

The median number of cumulative additional deaths, all ages, attributable to IPD over the first 5 years of a 1+1 schedule was 4 (UI, −1, 12), none of which were in children under 15 years of age. For pneumococcal CAP, the additional cumulative number was 4 (−1, 12), and 8 (−3, 23) with doubling of the pneumococcal CAP cases in 15+-year-olds—again, with none in children under 15 years of age (Table 4). Under the ‘0’+1 scenario, deaths increase to 13 (0, 32) for IPD, and for CAP, the increase is 11 (−1, 30) for the base case scenario and 21 (−3, 58) for doubling of the pneumococcal CAP cases in 15+-year-olds (S5 Table).

### Sensitivity analysis

Varying the duration of vaccine protection to 3 or 8 years revealed that the shorter duration would produce more IPD cases than a longer duration of protection compared with the base case of 5 years of protection when changing from 2+1 to 1+1 (S6 Table), although the overall increase in IPD cases would still only be 0.34% with the 3-year duration. The longer-term simulations out to 2030/2031 suggested that with a 3-year duration under the 2+1 schedule, VT2 cases would not be eliminated and that, in addition, under a 1+1 schedule, VT1 cases would also persist (S6 Fig). Results with exclusion of serotype 3 from the model are similar to those in the base case scenario, as presented in S6 Table.

## Discussion

We used a pneumococcal transmission model to investigate the likely impact of changing from a 2+1 to a 1+1 PCV13 schedule for infants in the UK. In this model, serotypes are grouped according to whether or not they are covered by the vaccine, with the key transmission parameters for the serotype groupings estimated by fitting to pre-PCV carriage and post-PCV IPD data. Based on the results of an immunogenicity study comparing post-booster responses after 1+1 and 2+1 schedules [7], we assumed a similar level of protection against carriage post-booster under the two schedules. We found that, with the current mature status of the UK PCV13 programme and the associated level of herd immunity, such a change would have little overall impact on the number of IPD or CAP cases in England and Wales over the next 5 years. The results of this modelling study informed the advice given by the UK Joint Committee on Vaccination and Immunisation (JCVI) that the UK should move to a 1+1 schedule for PCV13 [35].

Pneumococcal transmission involves more than 90 serotypes and is difficult to model due to insufficient knowledge of the interactions between individual serotypes. This necessitated the use of a relatively simplified compartmental model to simulate pneumococcal transmission dynamics and the impact of PCVs. Similar models were used to investigate the potential impact of PCV7 in the UK setting and, subsequently, of PCV13 [4,5,9]. With the exception of the recent rapid increase in NVT IPD, the model predictions have broadly reflected the post-PCV experience.

To investigate the larger-than-predicted increase in NVT IPD some 4 years after PCV13 introduction [12], we tested the hypothesis that LAIV administration to children might result in a short-lived increase in the FOI for pneumococcal transmission, irrespective of serotype. This seemed possible, as LAIV introduction coincided with the sharp increase in NVT and levelling off of the reduction in VT2 IPD, largely due to continuing cases of 19A [12]. The LAIV hypothesis failed to describe the rapid increase in NVT IPD cases from 2014/2015, even with an implausibly large (100%) increase in the FOI among LAIV vaccinated children (Fig 4). We tested two more hypotheses, namely that there was either an increase in FOI or an increase in CCR of the NVT group in 2014/2015, based on evidence from a PHE carriage study conducted in 2015/2016 [23]. Of these two, the increase in NVT CCR provided the best fit and was further explored by allowing the proportionate increase in the NVT CCR and the three competition parameters to vary by age in order to accommodate differences between age groups in the combination of serotypes comprising the NVT group. This improved the model fit with the most complex model, with six age groups having the smallest AIC value. This model showed large differences between age groups in the competition parameters and proportionate increase in NVT CCR (Table 2), consistent with complex age-dependent interactions between pneumococcal serotypes.

The simulation results indicated that changing the current 2+1 PCV13 schedule to a 1+1 schedule would be expected to have little overall impact on IPD cases in any age group within the next 5 years, with only a small increase in vaccine type IPD, which would be largely offset by a reduction in NVT cases. Although there would be reduced protection against VT carriage in the first year of life, when transmission probabilities per contact are highest (Fig 5), carriage protection in toddlers and preschoolers (cohorts in which transmission probabilities are still high) would be maintained under the 1+1 schedule by administration of the booster dose in the second year of life. Given the larger size of the toddler and preschool cohorts and their greater frequency of contacts than the infant cohort [16,17], their contribution to overall transmission in the population will be greater. This is consistent with the conclusions from ecological studies, which rely on the correspondence between time trends in reduction of VT IPD cases in adults and the decline in VT carriage prevalence post-PCV introduction in toddlers and preschoolers, and whose authors have also predicted little impact on herd immunity by changing to a 1+1 schedule [36].

One of our model assumptions is that the protection from two priming doses in infancy is the same as that from a booster dose in the second year of life, given after one or two infant priming doses, whereas evidence suggests that, for carriage, protection is higher for a booster dose [37]. Had we assumed lower protection against carriage for the two primary doses than the booster dose in the 2+1 schedule, then the impact of changing to a 1+1 schedule would have been less than predicted under our base case.

Our model predictions were robust in the sensitivity analysis to changes made to the assumed model parameter of duration of protection, and to whether serotype 3 was included as an NVT or entirely excluded from the model. While longer-term persistence of VT1 (PCV7) serotypes was predicted with a 3-year average duration of protection against carriage, a meta-analysis of PCV7 carriage studies indicated a mean duration of 6 years [38,39]. As expected, the worst-case scenario of a ‘0’+1 schedule, which assumed no protection against carriage acquisition or IPD from a single PCV13 dose in the first year of life, produced a greater increase in IPD cases (Table 3), with the slow re-establishment of endemic transmission of VT1 and VT2 in the decade after the schedule change (Figs 10b and 11b). While some studies do suggest poor protection against carriage from a single dose in infancy [40,41], there may still be reasonable protection against disease, as shown in post-licensure studies in England and Wales in which a single dose of PCV7 or PCV13 in the first year of life was estimated to provide 50%–60% protection [19,42] against IPD. However, this direct protection against IPD in infants would not mitigate the effects on herd immunity in older age groups if protection against carriage acquisition is truly zero with one infant dose. The UK JCVI therefore committed to review any change to IPD epidemiology subsequent to adoption of the 1+1 schedule and, if necessary, to act if the change was inconsistent with the model predictions [35].

The model predicts that despite the direct and indirect protection afforded by PCV7 and PCV13 against VT1 and VT2 serotypes, continued occurrence of VT IPD can be expected in young children until the mid-2020s, even continuing with the 2+1 schedule. Given the lower estimated effectiveness of PCV against VT2 than VT1, it is to be expected that these serotypes will largely comprise those in the VT2 group, of which 19A is currently the most prevalent. Due to serotype replacement, the overall number of IPD cases in 2016/2017 and incidence were similar to those before replacement of PCV7 with PCV13 [12], and despite levelling off of NVT IPD incidence predicted after 2020, the overall number is expected to continue to increase as the number of elderly people in the population grows (Fig 9). While this might suggest that PCV13 vaccination has brought no overall benefit, stopping the programme (the 0+0 scenario) would result in a large increase in cases within a decade, especially in children under 2 years old, in whom annual numbers of IPD cases are predicted to increase by more than 500 (Fig 10c). Under the current 2+1 programme, or with the change to a 1+1 schedule (S5 Fig), current reductions in the number of cases in under-65year-olds are expected to be maintained into the future. The absolute increase in cases in 65+-year-olds compared with the pre-PCV7 period reflects the sharply increasing IPD incidence with age in this group [43] and its progressively increasing mean age in future years [15].

The burden of pneumococcal disease is not limited to IPD and includes the more common disease presentation of non-bacteraemia pneumonia. While this could not be modelled, we made the assumption that any proportional change in IPD cases as a result of adopting a 1+1 schedule would also apply to pneumococcal-attributable CAP. However, a recent study showed no impact on pneumonia of unidentified cause in adults post-PCV, but rather an increase [28], which might reflect more aggressive replacement with NVTs in non-bacteraemia pneumonia than IPD in this age group. If so, a 1+1 schedule might have a beneficial effect on pneumococcal CAP in adults. Nevertheless, even assuming a similar impact to that predicted by the model on IPD, the additional cases of pneumococcal CAP would be small (UI on base case cumulative numbers over 5 years, −10, 242), with an associated mortality of 8 (−2, 24) additional deaths, including those attributable to IPD. Even doubling the pneumococcal CAP incidence in adults in the sensitivity analysis only produced a maximum of 35 additional deaths (CAP + IPD) over 5 years under the 1+1 schedule, none of which would be in children under 15 years of age (Table 4).

Our results contrast with those from a recent modelling study of the impact of a 1+1 schedule conducted by the manufacturer of PCV13 that predicted an additional 241 deaths over 5 years, the majority from pneumococcal CAP [44]. This model used no carriage data in model fitting but relied on estimates of CCRs from a study in under-2-year-olds conducted some years before PCV7 introduction in the Oxfordshire region of England. In the absence of other data, it was assumed that the CCR estimate for young children applied to all age groups. However, if this assumption were true, given the higher rates of IPD in 65+-year-olds than children, carriage prevalence would be highest in 65+-year-olds, whereas it is the lowest [45]. Furthermore, the NVT CCR estimate derived from the pre-PCV7 study in young children was around 10-fold lower than for vaccine serotypes. As a consequence, any reduction in NVT carriage as a result of an increase in VT carriage induced by a change to a 1+1 schedule would do little to offset the increase in VT IPD. In contrast, when estimated from our model fitting procedures, CCRs were highly age dependent, and the NVT CCR was higher than the VT2 CCR in adults, with a good fit between the model and recent NVT IPD cases. Therefore, any increase in VT2 carriage and IPD as a result of the change to a 1+1 schedule in our model would be offset by a greater reduction in NVT carriage and IPD in adults.

Despite the limited numbers of serotypes contained in PCV7 and PCV13, their reduction in carriage has greatly perturbed the pre-vaccination equilibrium within the nasopharynx. If sufficient data and understanding of how individual serotypes interact with each other were available, an individual-based model (IBM) would be an ideal modelling platform to describe such complex multi-strain dynamics underlying pneumococcal transmission. However, such an IBM would require much more detailed data than are currently available for all carried serotypes by age to understand their interactions, together with hugely increased computing power and time for fitting than with a deterministic model. Flasche and colleagues [46] and Cobey and Lipsitch [47] built hypothetical IBMs with 20 or 25 serotypes to study the coexistence and competition between pneumococcal serotypes in the population and incorporated serotype-specific and non-serotype-specific immunity after pneumococcal infection. Both IBMs could be used for measuring the impact of PCV programmes if there were ever sufficient data for them to be fitted. Nurhonen and colleagues [48] developed a simplified IBM that grouped pneumococcal serotypes into five groups (two PCV7 groups, PCV13–10-valent pneumococcal conjugate vaccine [PCV10; Synflorix, GSK], PCV10–PCV7, and a non-PCV13 serogroup). The PCV7 serotypes were subdivided into two groups according to the diversity of CCRs between serotypes. The PCV13 serotypes were subdivided, as the additional three serotypes (1, 5, and 7F) in PCV10 are less prevalent in carriage but more invasive than the extra three serotypes (3, 6A, and 19A) in PCV13. Despite developing a simplified version of the pneumococcal transmission IBM, the authors acknowledged that the lack of sufficiently detailed serotype-specific carriage data in all age classes would limit the applicability of their model.

Our compartmental model also has limitations. By grouping disparate serotypes such as NVTs together and estimating key transmission parameters for the group as a whole from the pre-PCV7 era, our model does not take account of newly emergent NVTs post-PCV that may have different transmission characteristics or invasiveness potential. While the 2015/2016 PHE carriage study [23] provided a justification for increasing the CCR of NVTs from 2014/2015, it does not throw light on why more invasive serotypes have emerged and whether this is vaccine driven or the result of other factors. This means that there is inherent uncertainty in the future behaviour of NVTs, which cannot be incorporated in the long-term simulations for which we assumed that the increased CCR of the NVT group post-PCV13 would continue in the future. While our model produced a good fit to VT and NVT cases in young children and overall, it overestimated the NVT cases in the post-PCV7 period in 5–64-year-olds and underestimated them in 65+-year-olds (S4 Fig). It also predicted a slower evolution of herd immunity for VT1 post-PCV7 than observed in the surveillance data. This suggests that the POLYMOD mixing matrix may not properly capture the contact patterns that underlie pneumococcal transmission in older age groups. Also, although the model predicts that the increase in NVT cases should level off after 2020 due to the low levels of VT1 and VT2 carriage, there may be unpredicted fluctuations due to changes in the incidence of co-circulating respiratory viruses [49], or secular changes in individual NVTs that are not vaccine driven, which cannot be accommodated in the model. Finally, our model and the fitting process were complex, with 26 fitted parameters, which, although improving the fit, may mean there are identifiability issues with some model parameters, and there were challenges in representing the uncertainty of our predictions, which meant we could not specify the exact coverage of our UI.

The results of our modelling study have relevance for other countries with mature PCV programmes and high vaccine coverage, and where pneumococcal transmission dynamics and mixing patterns are similar to those in England and Wales. PCV is an expensive vaccine, and reducing the number of doses given should reduce overall financial costs of the programme with, as indicated by our results, little loss of benefit. However, caution should be exercised in directly applying our results to similar settings, as local factors such as the timeliness of vaccination, particularly in administering the booster dose, will influence the outcome of a schedule change. In countries such as Africa, where carriage prevalence and the FOI is higher than in England and Wales, experience with use of PCVs is still limited, and the importance of a later booster dose both for individual and herd protection is not well documented. In such high-transmission settings, models such as ours can provide a theoretical framework for investigating the potential impact of different schedules and for helping to understand observed epidemiological trends after PCV introduction [50].

In conclusion, our simulations suggest that given the current mature status of the PCV programme in England and Wales, dropping one of the primary doses in the first year of life would have little impact on overall IPD control at any age. We estimate that reduction in the number of priming doses would improve programmatic efficiency, thereby facilitating the introduction of new vaccines by reducing the number of coadministered vaccines given at 2 and 4 months of age in the current UK schedule.

## Supporting information

S1 Data Data sets for the number of IPD cases from 2005/2006 to 2015/2016 (epidemiological years from 1 July to 30 June of subsequent calendar year) in England and Wales. Worksheets include (1) IPD data—ST3 in non-PCV13: ST3 IPD cases included with non-PCV13 serotype as a base case scenario; (2) IPD data—ST3 excluded: ST3 IPD cases excluded for a sensitivity analysis; and (3) 2001 carriage data: serotype distribution among the nasopharyngeal swabs collected in 2001/2002 in England by serogroupings and age groups, with two tables, depending on whether ST3 is included in NVT or excluded from the data. IPD, invasive pneumococcal disease; NVT, non-vaccine serotype; PCV13, 13-valent pneumococcal conjugate vaccine; ST3, serotype 3.
(XLSX)

S1 Fig Depiction of the waning function for protection against carriage acquisition (VEc) and disease (VEd). Theoretical plots showing waning protection against IPD (given carriage) and carriage acquisition after a booster or priming dose, with a 5-year duration of vaccine protection for full or partial protection and vaccine efficacy against carriage of 60% for the two priming doses or booster dose. Half the protection against carriage is assumed for one priming dose in infancy. Efficacy against IPD given carriage starts at 100%, irrespective of the number of doses, and declines with the same waning function as for vaccine efficacy against carriage. The red lines show overall protection levels, with a green line for full protection and blue line for partial protection. (A) Against IPD development with a booster dose, (B) against IPD development with a single priming dose, (C) against carriage acquisition with a booster dose, and (D) against carriage acquisition with a single priming dose. IPD, invasive pneumococcal disease; VEc, vaccine efficacy against carriage; VEd, vaccine efficacy against IPD.
(TIF)

S2 Fig LAIV coverage data. Annual uptake of LAIV among children in England and Wales between 2013/2014 and 2015/2016. These uptake data were used to fit the LAIV scenario in the main paper. Children over 5 years and under 9 years were vaccinated from 2016/2017 but were not included because the fitting period was until 2015/2016. LAIV, live attenuated influenza vaccine.
(TIF)

S3 Fig Model fitting flowchart for generating long-term simulations. (TIF)

S4 Fig Fitting results from the final model and data points by serotype groupings and age groups (A–F) and overall IPD cases (G) between 2005/2006 and 2015/2016 in England and Wales: Results show UI (range of values for the 500 accepted parameter sets), with each parameter set restricted to ±0.3 of maximum likelihood value. IPD, invasive pneumococcal disease; UI, uncertainty interval.
(TIF)

S5 Fig Long-term model predictions of annual IPD cases by age groups (A–F) in England and Wales between 2005/2006 and 2030/2031 and overall (G) with changing the current 2+1 PCV13 schedule to 1+1 in 2018: Results show the UI (range of 500 accepted parameter sets), with each parameter set restricted to ±0.3 of maximum likelihood value. IPD, invasive pneumococcal disease; PCV13, 13-valent pneumococcal conjugate vaccine; UI, uncertainty interval.
(TIF)

S6 Fig Long-term model predictions by serotype grouping, all ages, for a 2+1 (A) and 1+1 (B) schedule with a 3-year average duration of protection against carriage: Results show the UI (range of predictions based on the selected 1,000 parameter sets, with each parameter set restricted to ±0.05 of maximum likelihood value). UI, uncertainty interval.
(TIF)

S1 Text Model fitting and selection, and model prediction and uncertainty estimation. (DOCX)

S1 Table AIC values of the best fitted models of four scenarios considered and their best fit parameters, shown as a percentage increase from the ‘no assumption’ scenario. AIC, Akaike Information Criterion.
(DOCX)

S2 Table AIC values for models, assuming a proportional increase in NVT CCR from 2014/2015 in England and Wales. Four models allowed competition parameters to vary between different age groups while assuming a constant proportional increase in NVT CCR in all age groups. The last model assumed, in addition, an age-group dependent proportional increase in NVT CCR. AIC, Akaike Information Criterion; CCR, case-carrier ratio; NVT, non-vaccine serotype group.
(DOCX)

S3 Table Annual number of new carriage infections per 100,000 by serotype groupings and age group during the pre-PCV7 era in England and Wales estimated from a static model fitted to the longitudinal nasopharyngeal swab data collected in 2001/2002 in England [13]. PCV7, 7-valent pneumococcal conjugate vaccine.
(DOCX)

S4 Table CFRs for pneumococcal CAP and IPD by age groups. Pneumococcal CAP CFRs were obtained from Melegaro and colleagues [32] for under-44-year-olds and Luna and colleagues [34] for 45+-year-olds. CFRs for IPD were obtained from studies of laboratory confirmed cases in England, Wales, and Canada [29,30,31]. CAP, community-acquired pneumonia; CFR, case fatality rate; IPD, invasive pneumococcal disease.
(DOCX)

S5 Table Percentage change in accumulated overall IPD cases, incremental deaths due to IPDs, and potential incremental cases of, and deaths from, pneumococcal CAP for the first 5 years by age group when changing the current 2+1 schedule to ‘0’+1 in England and Wales in September 2018. Pneumonia with unknown organism (ICD-10 code J18) from Thorrington and colleagues [38], as used to calculate the pneumococcal CAP cases under 15 years, assuming 28% of J18 are pneumococcal attributable. In 15+-year-olds, pneumococcal CAP incidence by age group was obtained from the 2012/2013 pneumococcal CAP incidences in Table 2, Rodrigo and colleagues [26], and the annual cases were estimated using the population projection in England and Wales. We multiplied the pneumococcal CAP cases by the percentage change from 2+1 to ‘0’+1 to calculate the accumulated incremental cases due to the schedule change for the first 5 years. The sensitivity scenario presents the results when doubling the pneumococcal CAP incidence for those aged 15+ years from Rodrigo and colleagues. CAP, community-acquired pneumonia; IPD, invasive pneumococcal disease.
(DOCX)

S6 Table Sensitivity analyses. The tables below show differences in numbers of IPD cases for the first 5 years when changing the current 2+1 schedule to 1+1 in 2018, with three different average durations of PCV protection: (A) 3 years, (B) 5 years, (C) 8 years, and (D) excluding ST3 from the study and with 5 years’ average duration of protection. Results show the median of 1,000 accepted parameter sets and UI (range of accepted sets), with the range of each parameter set restricted to ±0.05 of maximum likelihood value. IPD, invasive pneumococcal disease; PCV, pneumococcal conjugate vaccine; ST3, serotype 3; UI, uncertainty interval.
(DOCX)

S1 Equation Pre-PCV static model. PCV, pneumococcal conjugate vaccine.
(DOCX)

S2 Equation Dynamic transmission model. (DOCX)

## Abbreviations

AIC: Akaike Information Criterion
CAP: community-acquired pneumonia
CCR: case-carrier ratio
CFR: case fatality rate
CRM: cross-reacting material
FOI: force of infection
IBM: individual-based model
IPD: invasive pneumococcal disease
JCVI: Joint Committee on Vaccination and Immunisation
LAIV: live attenuated influenza vaccine
NVT: non-vaccine serotype group with serotype 3
PCV: pneumococcal conjugate vaccine
PCV10: 10-valent pneumococcal conjugate vaccine
PCV13: 13-valent pneumococcal conjugate vaccine
PCV7: 7-valent pneumococcal conjugate vaccine
PHE: Public Health England
SIS: susceptible-infectious-susceptible
UI: uncertainty interval
VEc: vaccine efficacy against carriage
VEd: vaccine efficacy against IPD
VT1: vaccine serotype group 1 (PCV7 serotypes)
VT2: vaccine serotype group 2 (serotypes only in PCV13 not in PCV7 excluding 1 and 3)
