# Supplementary figures and images for: Estimated impact of revising the 13-valent pneumococcal conjugate vaccine schedule from 2+1 to 1+1 in England and Wales: A modelling study
Source: PLoS Med. 2019 Jul 3;16(7):e1002845. doi: 10.1371/journal.pmed.1002845 (PMC6608946; doi:10.1371/journal.pmed.1002845)

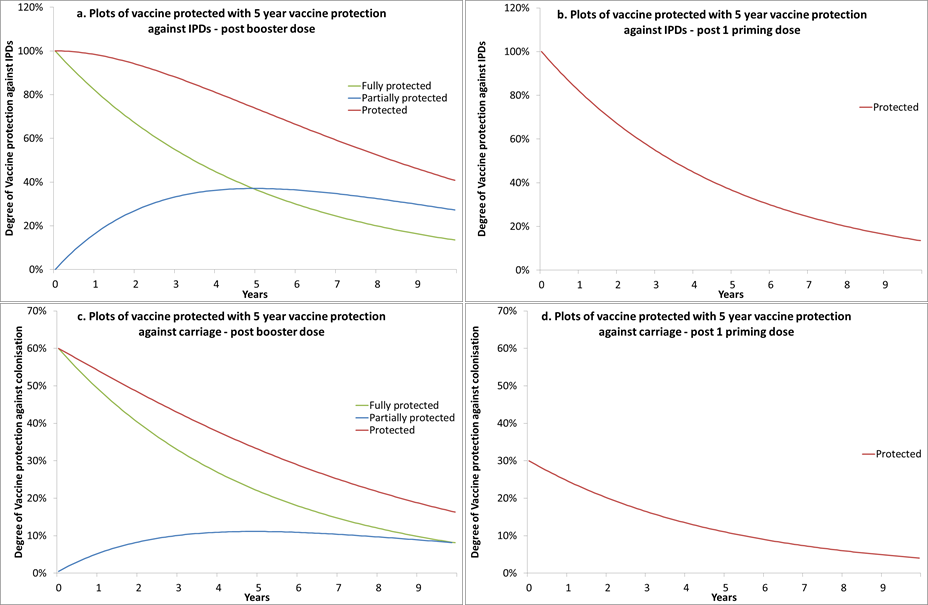

Supplement: S1 Fig — Theoretical plots showing waning protection against IPD (given carriage) and carriage acquisition after a booster or priming dose, with a 5-year duration of vaccine protection for full or partial protection and vaccine efficacy against carriage of 60% for the two priming doses or booster dose. Half the protection against carriage is assumed for one priming dose in infancy. Efficacy against IPD given carriage starts at 100%, irrespective of the number of doses, and declines with the same waning function as for vaccine efficacy against carriage. The red lines show overall protection levels, with a green line for full protection and blue line for partial protection. (A) Against IPD development with a booster dose, (B) against IPD development with a single priming dose, (C) against carriage acquisition with a booster dose, and (D) against carriage acquisition with a single priming dose. IPD, invasive pneumococcal disease; VEc, vaccine efficacy against carriage; VEd, vaccine efficacy against IPD. (TIF) [file pmed.1002845.s002.tif]

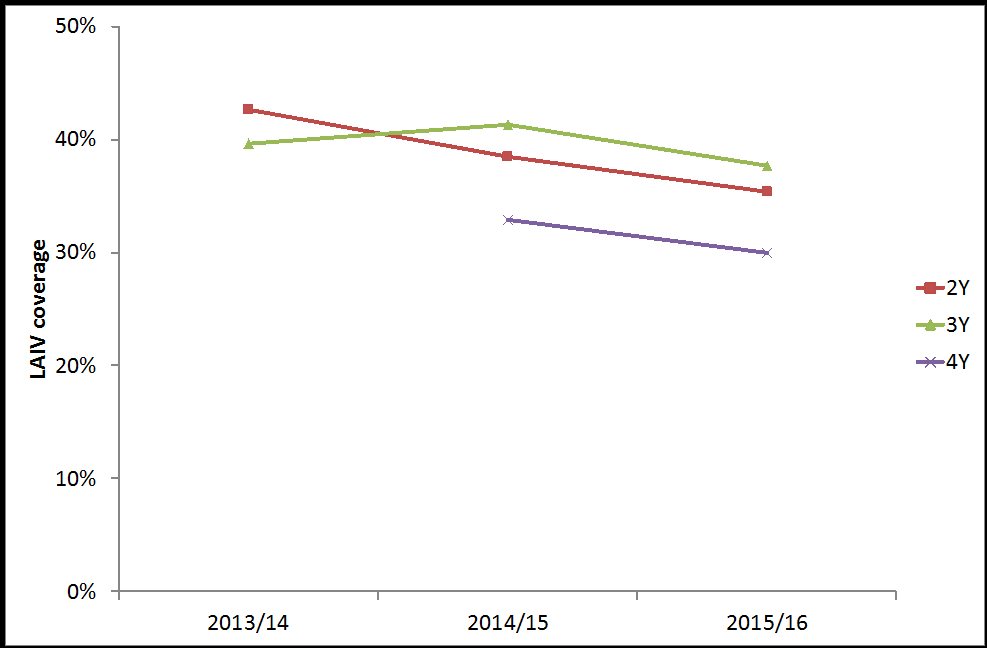

Supplement: S2 Fig — Annual uptake of LAIV among children in England and Wales between 2013/2014 and 2015/2016. These uptake data were used to fit the LAIV scenario in the main paper. Children over 5 years and under 9 years were vaccinated from 2016/2017 but were not included because the fitting period was until 2015/2016. LAIV, live attenuated influenza vaccine. (TIF) [file pmed.1002845.s003.tif]

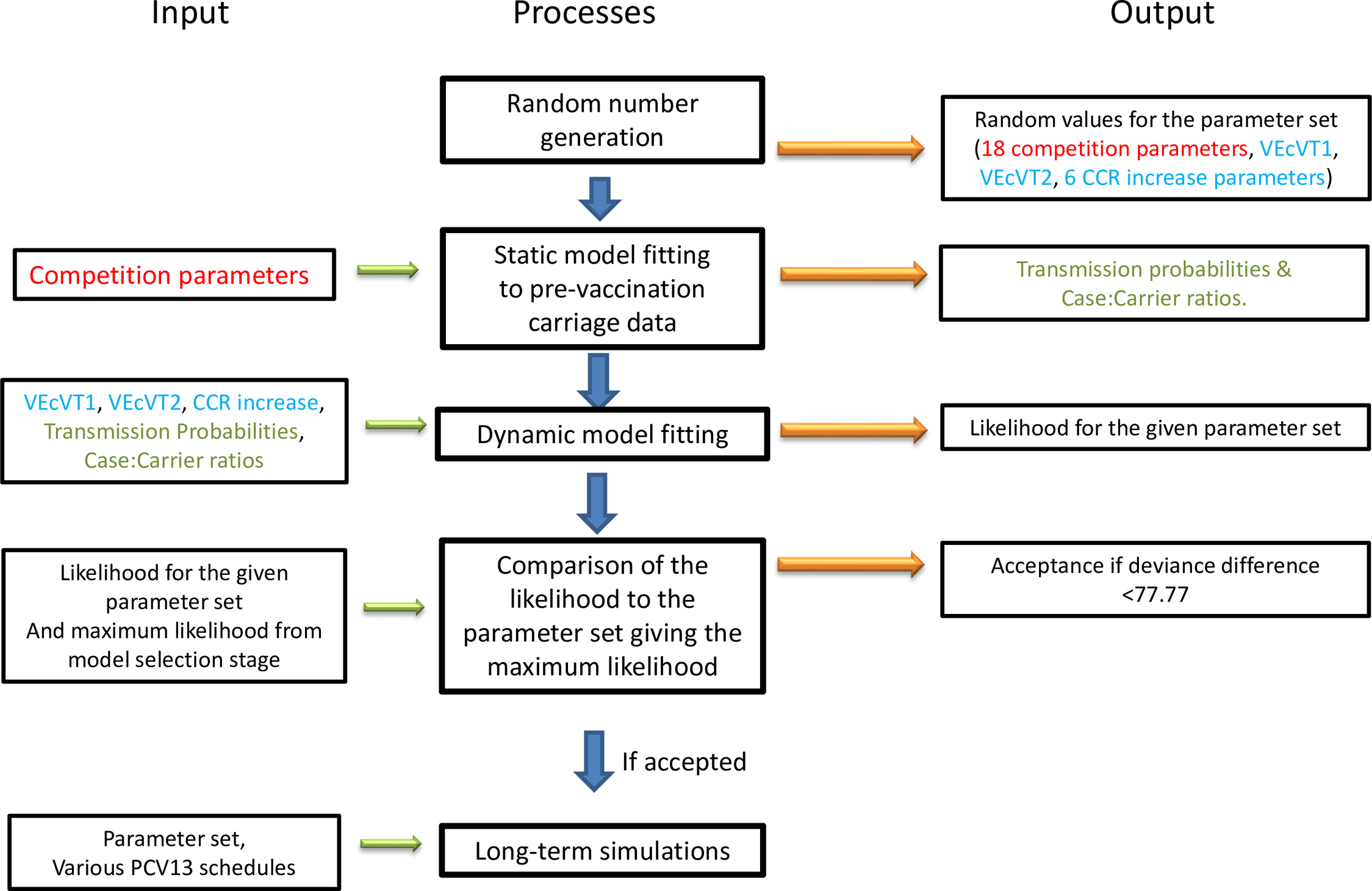

Supplement: S3 Fig — (TIF) [file pmed.1002845.s004.tif]

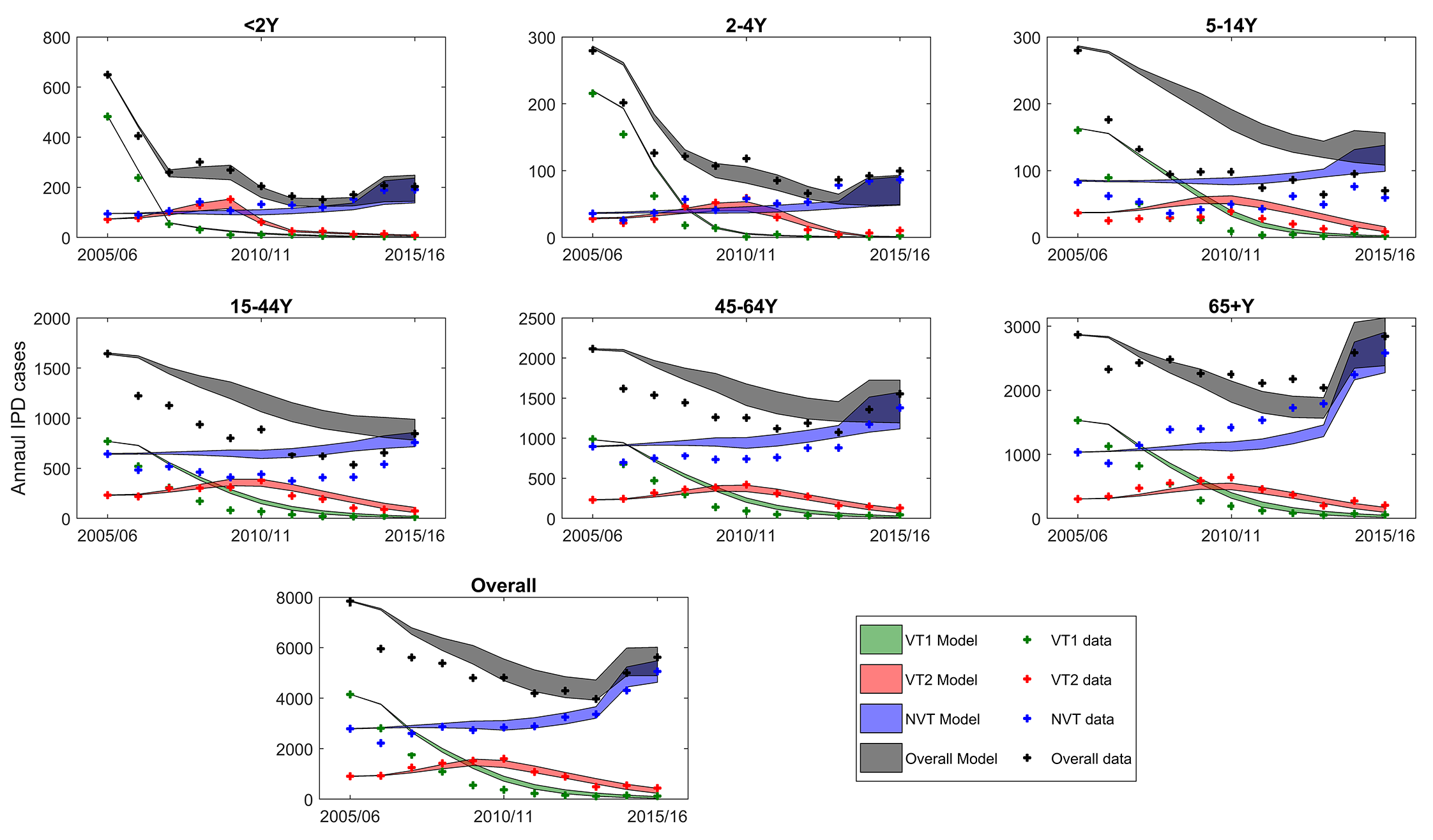

Supplement: S4 Fig — IPD, invasive pneumococcal disease; UI, uncertainty interval. (TIF) [file pmed.1002845.s005.tif]

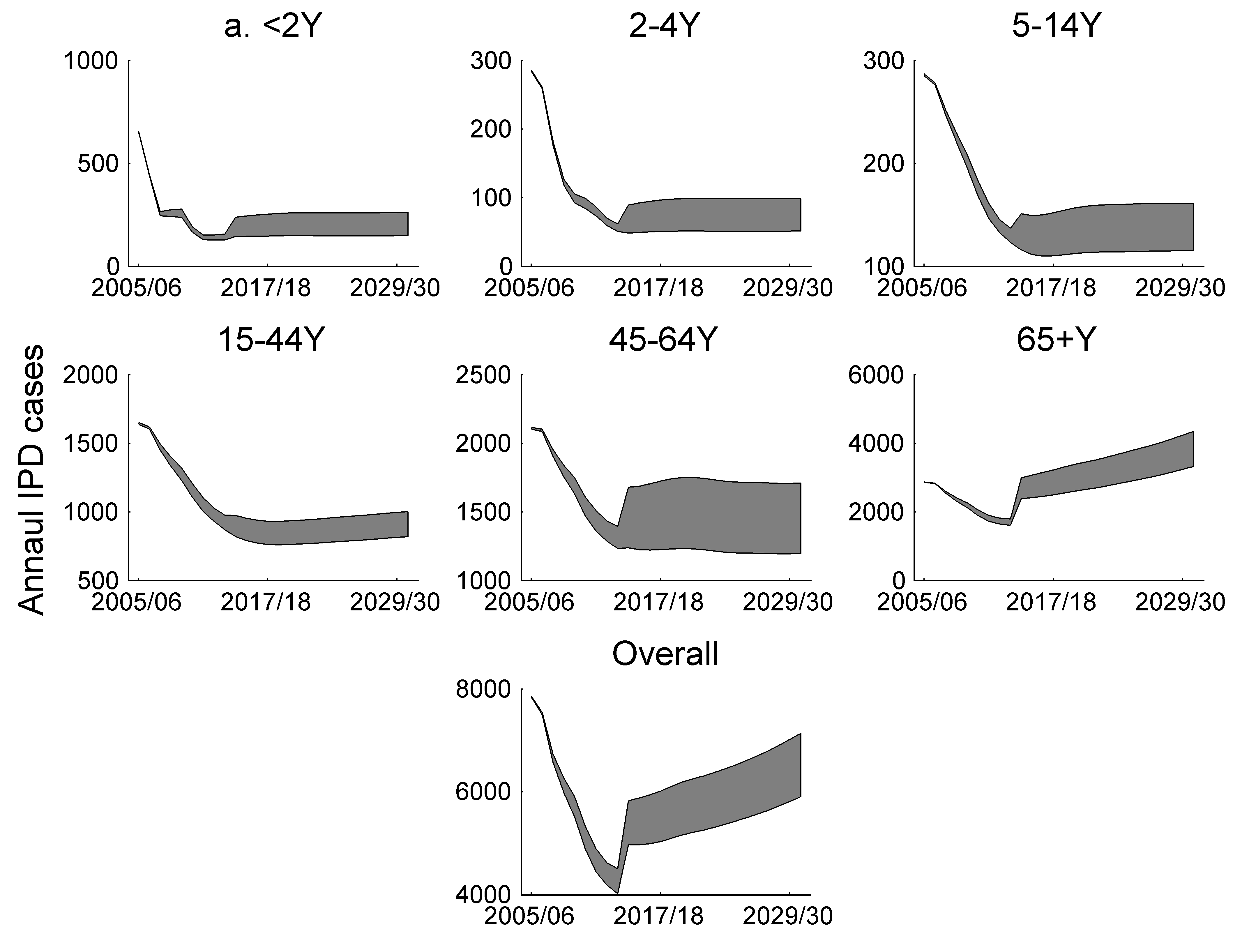

Supplement: S5 Fig — IPD, invasive pneumococcal disease; PCV13, 13-valent pneumococcal conjugate vaccine; UI, uncertainty interval. (TIF) [file pmed.1002845.s006.tif]

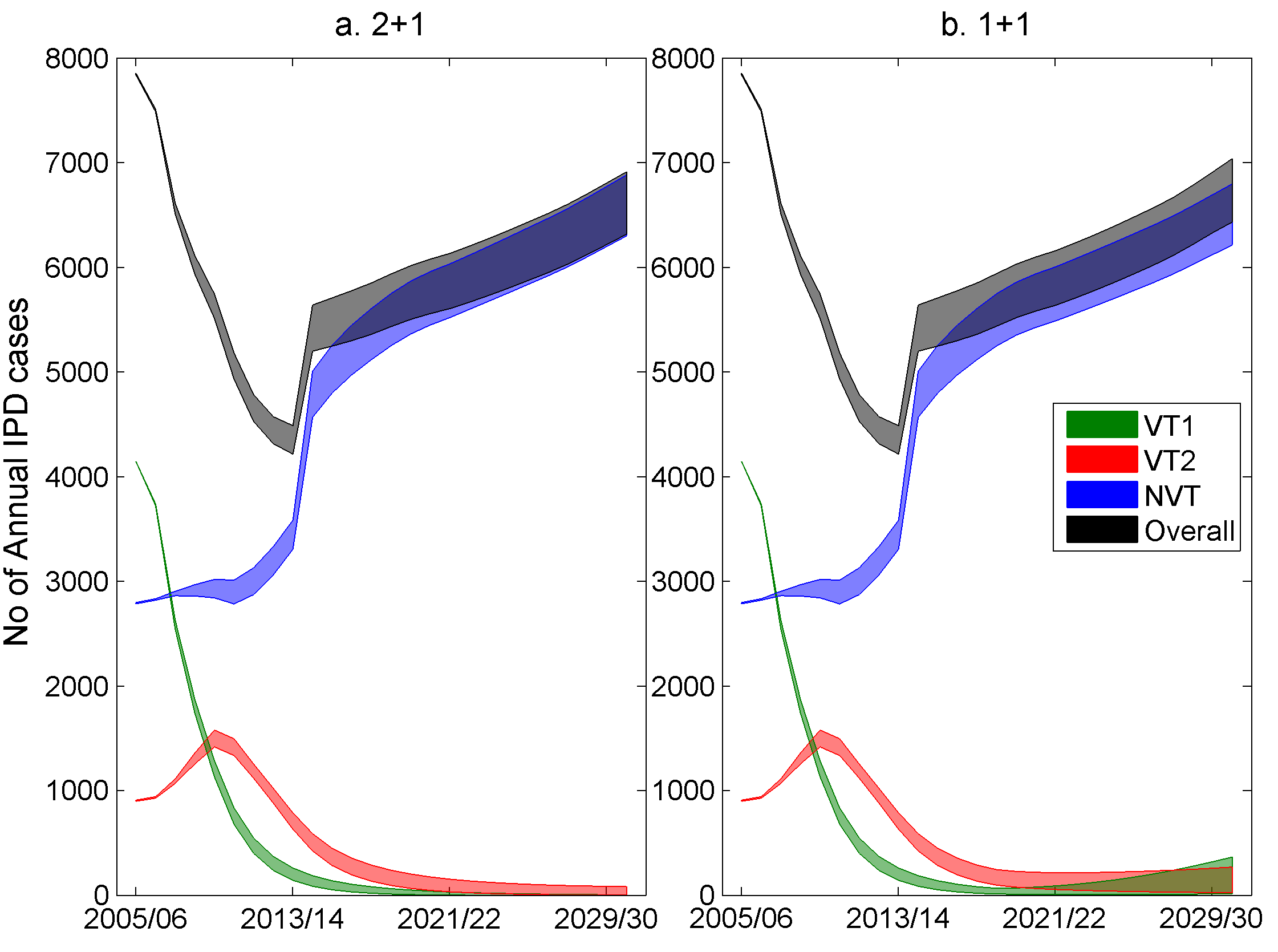

Supplement: S6 Fig — UI, uncertainty interval. (TIF) [file pmed.1002845.s007.tif]
