# Supplementary material for: Estimated impact of revising the 13-valent pneumococcal conjugate vaccine schedule from 2+1 to 1+1 in England and Wales: A modelling study
Source: PLoS Med. 2019 Jul 3;16(7):e1002845. doi: 10.1371/journal.pmed.1002845 (PMC6608946; doi:10.1371/journal.pmed.1002845)
Supplement: S1 Text — (DOCX) [file pmed.1002845.s008.docx]

## S1 Text:

## Model fitting and selection

The Nelder-Mead (Downhill Simplex) method was implemented to estimate the model parameters with the maximum Poisson likelihood by fitting to the pre-PCV7 carriage prevalence data and IPD data between 2005/06 and 2015/16 by three serogroups and age groups.  Model parameters consisted of competition parameters between serotype groupings (which in some models were age dependent), vaccine efficacies against acquisition of carriage of VT1 (VEcVT1) and VT2 (VEcVT2) serotypes, and a parameter (or in some models age specific parameters) to describe the rapid increase in the NVT IPD cases from 2014/15.  Fitting to obtain maximum likelihood estimates was then done in a two stage process. First a static model was fitted to the pre-PCV7 carriage prevalence data based on the competition parameters and this used to calculate transmission probabilities per contact and case:carrier ratios (CCR) by serotype grouping (VT1, VT2 and NVT) and age groups. In this step the Nelder-Mead method of maximizing the Poisson likelihood is actually used as numerical method for solving the static model to give the transmission probabilities and CCRs since an exact fit is always obtained (log-likelihood = 0) for a given set of competition parameters. Next the output from stage 1 along with the vaccine efficacies and the parameter(s) for the increase were included in the dynamic model that was fitted to the 2005/6-2015/16 IPD data.

To identify the maximum likelihood in the large parameter space 10 random sets of starting values were chosen. For the models without age specific competition parameters this produced consistent results, but for the model with age specific competition parameters different local maxima were found. For this model the parameter set with highest likelihood was chosen and this was refitted with these values as the starting values to further refine the estimates. For this model an additional check was done by comparing this maximum likelihood to the likelihoods obtained from the search of the parameter space used for the uncertainty bounds (see later section on model prediction and uncertainty estimation). Whilst this did give 5 sets (from many 1000s searched) with a marginally improved likelihood none of these would have had a large influence on the parameter space covered for identifying the 500 parameter sets for uncertainty of the predictions.

The fitting was conducted for various models according to the number of age groups for the competition parameters and proportional increase in NVT CCR from 2014/15 as described below.

First, one set of competition parameters for all age groups was used to select the best model among four scenarios to describe the rapid increase in NVT IPD cases since 2014/15. These were:

1. no assumption, pre-PCV7 FOIs and CCRs used without modification in the post-PCV period
2. a transient proportional increase in FOI (between 0 and 100%) for all serotypes for one month after receipt of LAIV (LAIV FOI) starting in November 2013/14 and reflecting the annual age-specific LAIV coverage
3. a proportional increase in NVT Force of Infection from 2014/15 (NVT FOI) which results in an increase in carriage prevalence for NVTs
4. a proportional increase in NVT CCR from 2014/15 which does not affect carriage prevalence just the probability of IPD given carriage

The Akaike Information Criteria (AIC) values of these four models indicated the best fitting model to be with the proportional increase in NVT CCR (S1 Table).

Next, we investigated whether the fitting could be improved by having age-dependent competition parameters and allowing the proportional increase in NVT CCR to vary by age group to reflect the different distributions of NVTs in each age group. We considered three sets of age groupings for this investigation:

1. Two age groups (<5Y and 65+ together, and 5-64Y)
2. Three age groups (<5Y, 5-64Y and 65+)
3. Six age groups (<2Y, 2-4Y, 5-14Y, 15-44Y, 45-64Y, and 65+Y)

The AIC values indicated that six age groups for both the proportional increase in NVT CCR and the three competition parameters gave the best fitting model (S2 Table). Furthermore the three competition parameters and proportional increase in NVT CCR were highly age dependent with little potential for collapsing age groups to allow model simplification (Table 2 in the main paper).

**Model prediction and uncertainty estimation**

To create uncertainty bounds for the final model, we randomly generated sets of model parameters until the procedure found sets with the difference between the deviance based on these fixed parameters and the deviance for the model with parameters with the maximum likelihood less than 77.77. This value of 77.77 is double 38.885 which is the 5% critical value from a chi-squared distribution with 26 degrees of freedom chosen to represent comparing a model with 26 fixed parameters to one allowing 26 parameters to vary. Doubling the critical value was done to compensate for over-dispersion in the data around the best fitting model (see Ladhani et al. [Lancet ID, 2018], footnote to table1 where over-dispersion in pre-PCV7 IPD rates was reported as 2.1). The searching routine was conducted in the High Performance Computing Cluster with 682 cores.

The steps involved in model fitting for the final model are shown in S2 Fig.

First in order to estimate the time required to find a single set of parameters satisfying the deviance criterion mentioned above, we tried the entire space for each parameter without any constraint (i.e allowing each to range randomly between 0 and 1) using a single processor but no parameter set was found despite running for 21 days. The parameter space was therefore incrementally constrained starting from a range of ±0.05 from the best fitted parameter values until the range of most of the accepted parameter values fell within the pre-specified range, stopping at 500 for the sets with the largest pre-specified range (±0.3). These 500 accepted sets of parameters are used to predict the potential long-term impact of the various PCV13 programmes considered under the base cases scenario of a 5 year average duration of protection. Due to the computing time to find acceptable parameter sets using the ±0.3 range, for the sensitivity analyses we narrowed the searching intervals to ±0.05 from the parameter set giving the maximum likelihood, which enabled 1,000 sets to be found. The sets of model parameters found for the 5 year average duration of protection with this smaller parameter interval gave similar results for the additional predicted cases under the change to a 1+1 schedule (S6 Table) to those obtained from the 500 sets of model parameters using the wider parameter intervals (Table 3).
