# Supplementary material for: Estimated impact of revising the 13-valent pneumococcal conjugate vaccine schedule from 2+1 to 1+1 in England and Wales: A modelling study
Source: PLoS Med. 2019 Jul 3;16(7):e1002845. doi: 10.1371/journal.pmed.1002845 (PMC6608946; doi:10.1371/journal.pmed.1002845)
Supplement: S1 Table — AIC, Akaike Information Criterion. (DOCX) [file pmed.1002845.s009.docx]

**S1 Table.** AIC values of the best fitted models of four scenarios considered and their best fit parameters, shown as a percentage increase from the ‘no assumption’ scenario.

| Scenarios | Parameters | Likelihood | AIC values of best fitted models | parameter value: % increase |
| --- | --- | --- | --- | --- |
| No assumption | 5 | -2887 | 5784 | - |
| LAIV FOI | 6 | -2819 | 5649 | 100% |
| NVT FOI | 6 | -2774 | 5560 | 3.3% |
| NVT CCR | 6 | -2721 | 5453 | 29.6% |

AIC, Akaike Information Criterion.
