# Supplementary material for: Estimated impact of revising the 13-valent pneumococcal conjugate vaccine schedule from 2+1 to 1+1 in England and Wales: A modelling study
Source: PLoS Med. 2019 Jul 3;16(7):e1002845. doi: 10.1371/journal.pmed.1002845 (PMC6608946; doi:10.1371/journal.pmed.1002845)
Supplement: S2 Table — Four models allowed competition parameters to vary between different age groups while assuming a constant proportional increase in NVT CCR in all age groups. The last model assumed, in addition, an age-group dependent proportional increase in NVT CCR. AIC, Akaike Information Criterion; CCR, case-carrier ratio; NVT, non-vaccine serotype group. (DOCX) [file pmed.1002845.s010.docx]

**S2 Table.** AIC values for models, assuming a proportional increase in NVT CCR from 2014/2015 in England and Wales. Four models allowed competition parameters to vary between different age groups while assuming a constant proportional increase in NVT CCR in all age groups. The last model assumed, in addition, an age-group dependent proportional increase in NVT CCR.

| Age groups for NVT CCR increase from 2014/15 | Age groups for competition parameters | No of Parameters | Likelihood | AIC |
| --- | --- | --- | --- | --- |
| 1 | 1 | 6 | -2744 | 5499 |
| 1 | 2 | 9 | -2559 | 5137 |
| 1 | 3 | 12 | -2551 | 5127 |
| 1 | 6 | 21 | -2472 | 4987 |
| 6 | 6 | 26 | -2188 | 4427 |

AIC, Akaike Information Criterion; CCR, case-carrier ratio; NVT, non-vaccine serotype.
