# Supplementary material for: Estimated impact of revising the 13-valent pneumococcal conjugate vaccine schedule from 2+1 to 1+1 in England and Wales: A modelling study
Source: PLoS Med. 2019 Jul 3;16(7):e1002845. doi: 10.1371/journal.pmed.1002845 (PMC6608946; doi:10.1371/journal.pmed.1002845)
Supplement: S3 Table — PCV7, 7-valent pneumococcal conjugate vaccine. (DOCX) [file pmed.1002845.s011.docx]

**S3 Table.** Annual number of new carriage infections per 100,000 by serotype groupings and age group during the pre-PCV7 era in England and Wales estimated from a static model fitted to the longitudinal nasopharyngeal swab data collected in 2001/2002 in England [13].

|  | 0Y | 1-2Y | 3-4Y | 5-9Y | 10-19Y | 20-39Y | 40+Y |
| --- | --- | --- | --- | --- | --- | --- | --- |
| VT1 | 247,342 (247,283, 247,409) | 212,841 (212,723, 212,946) | 219,903 (219,812, 219,972) | 180,334 (180,278, 180,349) | 108,382 (108,369, 108,392) | 85,695 (85,686, 85,702) | 91,285 (91,275, 91,303) |
| VT2 | 65,782 (62,085, 71,639) | 70,259 (68,601, 71,171) | 85,166 (81,219, 85,463) | 38,785 (38,042, 38,829) | 12,467 (12,368, 12,514) | 25,037 (24,829, 25,157) | 40,136 (39,880, 40,358) |
| NVT | 99,086 (92,665, 101,441) | 62,561 (60,915, 63,279) | 130,663 (125,548, 131,829) | 148,358 (145,620, 149,008) | 126,588 (125,937, 126,785) | 46,878 (46,582, 47,009) | 67,496 (67,098, 67,820) |

PCV7, 7-valent pneumococcal conjugate vaccine.
