# Supplementary material for: Estimated impact of revising the 13-valent pneumococcal conjugate vaccine schedule from 2+1 to 1+1 in England and Wales: A modelling study
Source: PLoS Med. 2019 Jul 3;16(7):e1002845. doi: 10.1371/journal.pmed.1002845 (PMC6608946; doi:10.1371/journal.pmed.1002845)
Supplement: S4 Table — Pneumococcal CAP CFRs were obtained from Melegaro and colleagues [32] for under-44-year-olds and Luna and colleagues [34] for 45+-year-olds. CFRs for IPD were obtained from studies of laboratory confirmed cases in England, Wales, and Canada [29,30,31]. CAP, community-acquired pneumonia; CFR, case fatality rate; IPD, invasive pneumococcal disease. (DOCX) [file pmed.1002845.s012.docx]

**S4 Table.** CFRs for pneumococcal CAP and IPD by age groups. Pneumococcal CAP CFRs were obtained from Melegaro and colleagues [32] for under-44-year-olds and Luna and colleagues [34] for 45+-year-olds. CFRs for IPD were obtained from studies of laboratory confirmed cases in England, Wales, and Canada [29,30,31].

|  | <2 | 2-4 | 5-14 | 15-44 | 45-64 | 65+ |
| --- | --- | --- | --- | --- | --- | --- |
| pneumococcal CAP | 0.33% | 0.21% | 0.28% | 3.01% | 4.60% | 10.70% |
| IPD | 4.80% | 4.80% | 1.80% | 9.33% | 12.45% | 26.59% |

CAP, community-acquired pneumonia; CFR, case fatality rate; IPD, invasive pneumococcal disease.
