# Supplementary material for: Estimated impact of revising the 13-valent pneumococcal conjugate vaccine schedule from 2+1 to 1+1 in England and Wales: A modelling study
Source: PLoS Med. 2019 Jul 3;16(7):e1002845. doi: 10.1371/journal.pmed.1002845 (PMC6608946; doi:10.1371/journal.pmed.1002845)
Supplement: S5 Table — Pneumonia with unknown organism (ICD-10 code J18) from Thorrington and colleagues [38], as used to calculate the pneumococcal CAP cases under 15 years, assuming 28% of J18 are pneumococcal attributable. In 15+-year-olds, pneumococcal CAP incidence by age group was obtained from the 2012/2013 pneumococcal CAP incidences in Table 2, Rodrigo and colleagues [26], and the annual cases were estimated using the population projection in England and Wales. We multiplied the pneumococcal CAP cases by the percentage change from 2+1 to ‘0’+1 to calculate the accumulated incremental cases due to the schedule change for the first 5 years. The sensitivity scenario presents the results when doubling the pneumococcal CAP incidence for those aged 15+ years from Rodrigo and colleagues. CAP, community-acquired pneumonia; IPD, invasive pneumococcal disease. (DOCX) [file pmed.1002845.s013.docx]

**S5 Table.** Percentage change in accumulated overall IPD cases, incremental deaths due to IPDs, and potential incremental cases of, and deaths from, pneumococcal CAP for the first 5 years by age group when changing the current 2+1 schedule to ‘0’+1 in England and Wales in September 2018. Pneumonia with unknown organism (ICD-10 code J18) from Thorrington and colleagues, as used to calculate the pneumococcal CAP cases under 15 years, assuming 28% of J18 are pneumococcal attributable. In 15+-year-olds, pneumococcal CAP incidence by age group was obtained from the 2012/2013 pneumococcal CAP incidences in Table 2, Rodrigo and colleagues, and the annual cases were estimated using the population projection in England and Wales. We multiplied the pneumococcal CAP cases by the percentage change from 2+1 to ‘0’+1 to calculate the accumulated incremental cases due to the schedule change for the first 5 years. The sensitivity scenario presents the results when doubling the pneumococcal CAP incidence for those aged 15+ years from Rodrigo and colleagues.

| Age group | Percentage Change in IPD cases | IPD deaths | Pneumococcal CAP | | | | | |
| --- | --- | --- | --- | --- | --- | --- | --- | --- |
|  |  |  | Base case | | | Sensitivity scenario | | |
|  |  |  | Cases | Incremental cases | Incremental deaths | Cases | Incremental cases | Incremental deaths |
| <2 | 3.19% (1.24%, 7.49%) | 2 (1, 3) | 1,143 | 182 (71, 428) | 1 (0, 1) | 1,143 | 182 (71, 428) | 1 (0, 1) |
| 2-4 | 0.02% (-0.33%, 0.35%) | 0 (0, 0) | 1,141 | 1 (-19, 20) | 0 (0, 0) | 1,141 | 1 (-19, 20) | 0 (0, 0) |
| 5-14 | 0.33% (0.09%, 0.79%) | 0 (0, 0) | 977 | 16 (4, 39) | 0 (0, 0) | 977 | 16 (4, 39) | 0 (0, 0) |
| 15-44 | 0.66% (0.26%, 1.43%) | 3 (1, 5) | 2,082 | 69 (27, 149) | 2 (1, 4) | 4,165 | 138 (55, 298) | 4 (2, 9) |
| 45-64 | 0.31% (0.08%, 0.71%) | 3 (1, 6) | 2,387 | 37 (10, 84) | 2 (0, 4) | 4,773 | 74 (19, 168) | 3 (1, 8) |
| 65+ | 0.14% (-0.06%, 0.44%) | 6 (-3, 18) | 8,442 | 59 (-26, 187) | 6 (-3, 20) | 16,883 | 118 (-52, 374) | 13 (-6, 40) |
| All | 0.37% (0.12%, 0.80%) | 13 (0, 32) | 16,171 | 365 (67, 907) | 11 (-1, 30) | 29,082 | 530 (78, 1327) | 21 (-3, 58) |

CAP, community-acquired pneumonia; IPD, invasive pneumococcal disease.
