# Supplementary material for: Estimated impact of revising the 13-valent pneumococcal conjugate vaccine schedule from 2+1 to 1+1 in England and Wales: A modelling study
Source: PLoS Med. 2019 Jul 3;16(7):e1002845. doi: 10.1371/journal.pmed.1002845 (PMC6608946; doi:10.1371/journal.pmed.1002845)
Supplement: S6 Table — The tables below show differences in numbers of IPD cases for the first 5 years when changing the current 2+1 schedule to 1+1 in 2018, with three different average durations of PCV protection: (A) 3 years, (B) 5 years, (C) 8 years, and (D) excluding ST3 from the study and with 5 years’ average duration of protection. Results show the median of 1,000 accepted parameter sets and UI (range of accepted sets), with the range of each parameter set restricted to ±0.05 of maximum likelihood value. IPD, invasive pneumococcal disease; PCV, pneumococcal conjugate vaccine; ST3, serotype 3; UI, uncertainty interval. (DOCX) [file pmed.1002845.s014.docx]

**S6 Table. Sensitivity analyses.** The tables below show differences in numbers of IPD cases for the first 5 years when changing the current 2+1 schedule to 1+1 in 2018, with three different average durations of PCV protection: (A) 3 years, (B) 5 years, (C) 8 years, and (D) excluding ST3 from the study and with 5 years’ average duration of protection. Results show the median of 1,000 accepted parameter sets and UI (range of accepted sets), with the range of each parameter set restricted to ±0.05 of maximum likelihood value.

(A) 3 years

|  | <2 | 2-4 | 5-14 | 15-44 | 45-64 | 65+ | Total |
| --- | --- | --- | --- | --- | --- | --- | --- |
| VT1 | 5 (2, 11) | 1 (0, 3) | 2 (1, 6) | 15 (5, 36) | 21 (7, 49) | 40 (12, 93) | 84 (27, 198) |
| VT2 | 9 (5, 15) | 1 (1, 2) | 4 (2, 6) | 33 (18, 51) | 36 (20, 57) | 57 (32, 90) | 140 (78, 222) |
| NVT | -7 (-11, -4) | -1 (-2, -1) | -2 (-3, -1) | -17 (-25, -10) | -30 (-45, -17) | -67 (-98, -39) | -124 (-182, -73) |
| **Overall** | **7 (3, 13)** | **1 (0, 3)** | **4 (3, 7)** | **31 (19, 51)** | **27 (14, 52)** | **30 (5, 75)** | **101 (47, 198)** |

(B) 5 years

|  | <2 | 2-4 | 5-14 | 15-44 | 45-64 | 65+ | Total |
| --- | --- | --- | --- | --- | --- | --- | --- |
| VT1 | 1 (0, 2) | 0 (0, 0) | 0 (0, 1) | 3 (1, 7) | 4 (1, 9) | 7 (2, 17) | 14 (5, 36) |
| VT2 | 5 (2, 9) | 0 (0, 1) | 2 (1, 3) | 18 (9, 32) | 20 (10, 35) | 31 (15, 55) | 74 (36, 134) |
| NVT | -4 (-7, -2) | -1 (-1, 0) | -1 (-2, -1) | -9 (-16, -5) | -16 (-28, -8) | -35 (-61, -18) | -65 (-115, -34) |
| **Overall** | **2 (0, 3)** | **0 (-1, 0)** | **1 (0, 1)** | **11 (6, 19)** | **8 (3, 14)** | **3 (-4, 13)** | **24 (9, 47)** |

(C) 8 years:

|  | <2 | 2-4 | 5-14 | 15-44 | 45-64 | 65+ | Total |
| --- | --- | --- | --- | --- | --- | --- | --- |
| VT1 | 0 (0, 1) | 0 (0, 0) | 0 (0, 0) | 1 (0, 2) | 1 (0, 3) | 2 (1, 5) | 4 (1, 11) |
| VT2 | 3 (1, 6) | 0 (0, 0) | 1 (0, 2) | 12 (5, 24) | 13 (6, 26) | 21 (9, 40) | 51 (21, 97) |
| NVT | -3 (-5, -1) | 0 (-1, 0) | -1 (-1, 0) | -6 (-11, -3) | -11 (-20, -5) | -24 (-45, -11) | -46 (-83, -21) |
| **Overall** | **1 (0, 2)** | **0 (-1, 0)** | **0 (0, 0)** | **7 (3, 13)** | **3 (1, 8)** | **-1 (-6, 3)** | **10 (3, 22)** |

(D) ST3 exclusion with 5 years average duration of protection

|  | <2 | 2-4 | 5-14 | 15-44 | 45-64 | 65+ | Total |
| --- | --- | --- | --- | --- | --- | --- | --- |
| VT1 | 1 (0, 2) | 0 (0, 0) | 0 (0, 1) | 2 (1, 7) | 3 (1, 9) | 6 (2, 17) | 13 (5, 36) |
| VT2 | 4 (2, 8) | 0 (0, 0) | 1 (1, 3) | 16 (7, 30) | 17 (8, 33) | 27 (12, 53) | 66 (30, 128) |
| NVT | -3 (-6, -1) | -1 (-1, 0) | -1 (-1, 0) | -7 (-13, -3) | -12 (-23, -6) | -25 (-48, -12) | -49 (-93, -22) |
| **Overall** | **2 (1, 4)** | **0 (-1, 0)** | **1 (0, 2)** | **11 (6, 20)** | **9 (4, 17)** | **9 (3, 19)** | **32 (15, 57)** |

IPD, invasive pneumococcal disease; PCV, pneumococcal conjugate vaccine; ST3, serotype 3; UI, uncertainty interval.
