# Supplementary material for: Estimated impact of revising the 13-valent pneumococcal conjugate vaccine schedule from 2+1 to 1+1 in England and Wales: A modelling study
Source: PLoS Med. 2019 Jul 3;16(7):e1002845. doi: 10.1371/journal.pmed.1002845 (PMC6608946; doi:10.1371/journal.pmed.1002845)
Supplement: S1 Equation — PCV, pneumococcal conjugate vaccine. (DOCX) [file pmed.1002845.s015.docx]

**S1 Equation:**

**Pre-PCV static model**

This is a static model of the pneumococcal compartments built to estimate the force of infections (FOI) by serogroups (VT1, VT2 and NVT) and age groups (0, 1-2, 3-4, 5-9, 10-19, 20-39 and 40Y+) at the pre-vaccination equilibrium.

**Equations S1.**

$$S_{i}=\left( 1-\lambda_{1,i}-\lambda_{2,i}-\lambda_{3,i} \right)S_{i-1}+\rho_{i-1}\left( {VT1}_{i-1}+{VT2}_{i-1}+{NVT}_{i-1} \right),$$

$${VT1}_{i}=\left( 1-\rho_{i-1}-\pi_{1,i}\lambda_{2,i}-{\pi_{2,i}\lambda}_{3,i} \right){VT1}_{i-1}+\rho_{i-1}\left( {VT1VT2}_{i-1}+VT1{NVT}_{i-1} \right),$$

$${VT2}_{i}=\left( 1-\rho_{i-1}-\pi_{4,i}\lambda_{1,i}-{\pi_{2,i}\lambda}_{3,i} \right){VT2}_{i-1}+\rho_{i-1}\left( {VT1VT2}_{i-1}+VT2{NVT}_{i-1} \right),$$

$${NVT}_{i}=\left( 1-\rho_{i-1}-\pi_{5,i}\lambda_{1,i}-{\pi_{6,i}\lambda}_{2,i} \right){NVT}_{i-1}+\rho_{i-1}\left( {VT1NVT}_{i-1}+VT2{NVT}_{i-1} \right),$$

$${VT1VT2}_{i}=\left( 1-{2\rho}_{i-1}-{\pi_{7,i}\lambda}_{3,i} \right){VT1VT2}_{i-1}+\pi_{1,i}\lambda_{2,i}{VT1}_{i-1}+\pi_{4,i}\lambda_{1,i}{VT2}_{i-1}+\rho_{i-1}{ALL}_{i-1,}$$

$${VT1NVT}_{i}=\left( 1-{2\rho}_{i-1}-{\pi_{8,i}\lambda}_{2,i} \right){VT1NVT}_{i-1}+\pi_{2,i}\lambda_{3,i}{VT1}_{i-1}+\pi_{5,i}\lambda_{1,i}{NVT}_{i-1}+\rho_{i-1}{ALL}_{i-1},$$

$${VT2NVT}_{i}=\left( 1-{2\rho}_{i-1}-{\pi_{9,i}\lambda}_{1,i} \right){VT2NVT}_{i-1}+\pi_{3,i}\lambda_{3,i}{VT1}_{i-1}+\pi_{6,i}\lambda_{2,i}N{VT}_{i-1}+\rho_{i-1}{ALL}_{i-1,}$$

${ALL}_{i}=\left( 1-{3\rho}_{i-1} \right){ALL}_{i-1}+\pi_{7,i}\lambda_{3,i}{VT1VT2}_{i-1}+\pi_{8,i}\lambda_{2,i}VT1N{VT}_{i-1}+\pi\lambda_{1,i}VT2N{VT}_{i-1}$,

for $i=2,\ldots,4800$ age cohorts (48 cohorts for each annual age cohort comprising 100 year cohorts between 0y and 99y) , where for $S_{1}=1,{VT1}_{1}={VT2}_{1}={NVT}_{1}={VT1VT2}_{1}={VT1NVT}_{1}={VT2NVT}_{1}={ALL}_{1}=0$, $\pi$ is a reduction parameter on the FOI, $\lambda,$ which is 1- Competition parameter between serogroups, and $\rho$ is a clearance rate, 1/ duration of colonisation.

The prevalence by serogroups and age groups are calculated as follows:

$$\mathrm{Prevalence}_{VT1,Age group}=\frac{\sum_{i\in Age group} \left( {VT1}_{i}{+VT1VT2}_{i}+VT1{NVT}_{i}+{ALL}_{i} \right)Population\left( Age \right)}{48*Population(Age group)},$$

$$\mathrm{Prevalence}_{VT2,Age group}=\frac{\sum_{i\in Age group} \left( {VT2}_{i}+{VT2NVT}_{i} \right)Population\left( Age \right)}{48*Population(Age group)},$$

$$\mathrm{Prevalence}_{NVT,Age group}=\frac{\sum_{i\in Age group} \left( {NVT}_{i} \right)Population\left( Age \right)}{48*Population(Age group)},$$

where assuming the detection rate for VT1 among co-infected compartment is 100%, and 100% for VT2 among VT2 and VT2NVT. This assumption could be changed if more sensitive serotyping method than quelling method is used to detect multiple infections in carriage samples. (Age = integer of (i/48), Population(i) = Population(Age)/48 as a “weekly” population size)

The Nelder-Mead (Downhill Simplex) method finds the best fitting model parameters for prevalence by maximising the following Poisson likelihood:

$LogLikelihood(Model|data) = \sum_{i=1}^{3} \sum_{j=1}^{7} \left( {CarriageData}_{i,j}*\log(\mathrm{Prevalence}_{i,j}*{CarriageData}_{all,j})-\mathrm{Prevalence}_{i,j}*{CarriageData}_{all,j} \right)$,

Where *i* is for three serogroups and *j* for seven age groups, and ${CarriageData}_{i,j}$ consists of positive swabs in total swabs tested (${CarriageData}_{all,j}$).

Once the static model is fitted to the pre-vaccination prevalence, the transmission probabilities per contact and Case:Carrier ratios by three serogroups and age groups are calculated. The equilibrium of the static model is used as the initial values for the unvaccinated group in the dynamic transmission model in order to minimise the computing time of the fitting and long-term simulation procedures.
