# Supplementary material for: Estimated impact of revising the 13-valent pneumococcal conjugate vaccine schedule from 2+1 to 1+1 in England and Wales: A modelling study
Source: PLoS Med. 2019 Jul 3;16(7):e1002845. doi: 10.1371/journal.pmed.1002845 (PMC6608946; doi:10.1371/journal.pmed.1002845)
Supplement: S2 Equation — (DOCX) [file pmed.1002845.s016.docx]

**S2 Equation:**

**Dynamic transmission model**

These are difference equations of the dynamic model of the pneumococcal transmission between serogroups, vaccination efficacy parameters against colonisation of vaccine serogroups and a potential parameter ($\alpha_{i}$) to describe the rapid increase in NVT IPD cases since 2014/15.

**Unvaccinated**:

$$S_{i,1}\left( t \right)=\left( 1-\lambda_{1,i}\left( t-1 \right)-\lambda_{2,i}\left( t-1 \right)-\lambda_{3,i}\left( t-1 \right)-\varphi_{0,i}(t-1) \right)S_{i-1,1}\left( t-1 \right)+\rho_{i-1}\left( {VT1}_{i-1,1}\left( t-1 \right)+{VT2}_{i-1,1}\left( t-1 \right)+{NVT}_{i-1,1}\left( t-1 \right) \right),$$

$${VT1}_{i,1}\left( t \right)=\left( 1-\rho_{i-1}-\pi_{1,i}\lambda_{2,i}\left( t-1 \right)-{\pi_{2,i}\lambda}_{3,i}\left( t-1 \right)-\varphi_{0,i}(t-1) \right){VT1}_{i-1,1}\left( t-1 \right)+\lambda_{1,i}\left( t-1 \right)S_{i-1,1}\left( t-1 \right)+\rho_{i-1}\left( {VT1VT2}_{i-1,1}\left( t-1 \right)+VT1{NVT}_{i-1,1}\left( t-1 \right) \right),$$

$${VT2}_{i,1}\left( t \right)=\left( 1-\rho_{i-1}-\pi_{4,i}\lambda_{1,i}\left( t-1 \right)-{\pi_{2,i}\lambda}_{3,i}\left( t-1 \right)-\varphi_{0,i}(t-1) \right){VT2}_{i-1,1}\left( t-1 \right)+\lambda_{2,i}\left( t-1 \right)S_{i-1,1}\left( t-1 \right)+\rho_{i-1}\left( {VT1VT2}_{i-1,1}\left( t-1 \right)+VT2{NVT}_{i-1,1}\left( t-1 \right) \right),$$

$${NVT}_{i,1}\left( t \right)=\left( 1-\rho_{i-1}-\pi_{5,i}\lambda_{1,i}\left( t-1 \right)-{\pi_{6,i}\lambda}_{2,i}\left( t-1 \right)-\varphi_{0,i}(t-1) \right){NVT}_{i-1,1}\left( t-1 \right)+\lambda_{3,i}\left( t-1 \right)S_{i-1,1}\left( t-1 \right)+\rho_{i-1}\left( {VT1NVT}_{i-1,1}\left( t-1 \right)+VT2{NVT}_{i-1,1}\left( t-1 \right) \right),$$

$${VT1VT2}_{i,1}\left( t \right)=\left( 1-{2\rho}_{i-1}-{\pi_{7,i}\lambda}_{3,i}\left( t-1 \right)-\varphi_{0,i}(t-1) \right){VT1VT2}_{i-1,1}\left( t-1 \right)+\pi_{1,i}\lambda_{2,i}{VT1}_{i-1,1}\left( t-1 \right)+\pi_{4,i}\lambda_{1,i}{VT2}_{i-1,1}\left( t-1 \right)+\rho_{i-1}{ALL}_{i-1,1}\left( t-1 \right),$$

$${VT1NVT}_{i,1}\left( t \right)=\left( 1-{2\rho}_{i-1}-{\pi_{8,i}\lambda}_{2,i}\left( t-1 \right)-\varphi_{0,i}(t-1) \right){VT1NVT}_{i-1,1}\left( t-1 \right)+\pi_{2,i}\lambda_{3,i}\left( t-1 \right){VT1}_{i-1,1}\left( t-1 \right)+\pi_{5,i}\lambda_{1,i}{\left( t-1 \right)NVT}_{i-1,1}\left( t-1 \right)+\rho_{i-1}{ALL}_{i-1,1}\left( t-1 \right),$$

$${VT2NVT}_{i,1}\left( t \right)=\left( 1-{2\rho}_{i-1}-{\pi_{9,i}\lambda}_{1,i}\left( t-1 \right)-\varphi_{0,i}(t-1) \right){VT2NVT}_{i-1,1}\left( t-1 \right)+\pi_{3,i}\lambda_{3,i}{VT1}_{i-1,1}\left( t-1 \right)+\pi_{6,i}\lambda_{2,i}N{VT}_{i-1,1}\left( t-1 \right)+\rho_{i-1}{ALL}_{i-1,1}\left( t-1 \right),$$

$${ALL}_{i,1}\left( t \right)=\left( 1-{3\rho}_{i-1}-\varphi_{0,i}(t-1) \right){ALL}_{i-1,1}\left( t-1 \right)+\pi_{7,i}\lambda_{3,i}\left( t-1 \right){VT1VT2}_{i-1,1}\left( t-1 \right) +\pi_{8,i}\lambda_{2,i}\left( t-1 \right)VT1N{VT}_{i-1,1}\left( t-1 \right)+\pi\lambda_{1,i}\left( t-1 \right)VT2N{VT}_{i-1,1}\left( t-1 \right),$$

**PCV7 partially protected**:

$$S_{i,2}\left( t \right)=\left( 1-\left( 1-d_{7}/2 \right)\lambda_{1,i}\left( t-1 \right)-\lambda_{2,i}\left( t-1 \right)-\lambda_{3,i}\left( t-1 \right)-\varphi_{1,i}(t-1)-\varphi_{2,i}\left( t-1 \right) \right)S_{i-1,2}\left( t-1 \right)+\rho_{i-1}\left( {VT1}_{i-1,2}\left( t-1 \right)+{VT2}_{i-1,2}\left( t-1 \right)+{NVT}_{i-1,2}\left( t-1 \right) \right)+\varphi_{0,i(if age<1)}\left( t-1 \right)S_{i-1,1}\left( t-1 \right)+\omega S_{i-1,3}\left( t-1 \right),$$

$${VT1}_{i,2}\left( t \right)=\left( 1-\rho_{i-1}-\pi_{1,i}\lambda_{2,i}\left( t-1 \right)-{\pi_{2,i}\lambda}_{3,i}\left( t-1 \right)-\varphi_{1,i}(t-1)-\varphi_{2,i}(t-1) \right){VT1}_{i-1,2}\left( t-1 \right)+\rho_{i-1}\left( {VT1VT2}_{i-1,2}\left( t-1 \right)+VT1{NVT}_{i-1,2}\left( t-1 \right) \right)+\left( 1-d_{7}/2 \right)\lambda_{1,i}\left( t-1 \right)S_{i-1,2}\left( t-1 \right)+\varphi_{0,i(if age<1)}\left( t-1 \right){VT1}_{i-1,1}\left( t-1 \right)+\omega{VT1}_{i-1,3}\left( t-1 \right),$$

$${VT2}_{i,2}\left( t \right)=\left( 1-\rho_{i-1}-\pi_{4,i}\left( 1-d_{7}/2 \right)\lambda_{1,i}\left( t-1 \right)-{\pi_{2,i}\lambda}_{3,i}\left( t-1 \right)-\varphi_{1,i}(t-1)-\varphi_{2,i}\left( t-1 \right) \right){VT2}_{i-1,2}\left( t-1 \right)+\lambda_{2,i}\left( t-1 \right)S_{i-1,2}\left( t-1 \right)+\rho_{i-1}\left( {VT1VT2}_{i-1,2}\left( t-1 \right)+VT2{NVT}_{i-1,2}\left( t-1 \right) \right)+\varphi_{0,i(if age<1)}\left( t-1 \right){VT2}_{i-1,1}\left( t-1 \right)+\omega{VT2}_{i-1,3}\left( t-1 \right),$$

$${NVT}_{i,2}\left( t \right)=\left( 1-\rho_{i-1}-\pi_{5,i}{\left( 1-d_{7}/2 \right)\lambda}_{1,i}\left( t-1 \right)-{\pi_{6,i}\lambda}_{2,i}\left( t-1 \right)-\varphi_{1,i}(t-1)-\varphi_{2,i}\left( t-1 \right) \right){NVT}_{i-1,2}\left( t-1 \right)+\lambda_{3,i}\left( t-1 \right)S_{i-1,2}\left( t-1 \right)+\rho_{i-1}\left( {VT1NVT}_{i-1,2}\left( t-1 \right)+VT2{NVT}_{i-1,2}\left( t-1 \right) \right)+\varphi_{0,i(if age<1)}\left( t-1 \right){NVT}_{i-1,1}\left( t-1 \right)+\omega{NVT}_{i-1,3}\left( t-1 \right),$$

$${VT1VT2}_{i,2}\left( t \right)=\left( 1-{2\rho}_{i-1}-{\pi_{7,i}\lambda}_{3,i}\left( t-1 \right)-\varphi_{1,i}(t-1)-\varphi_{2,i}(t-1) \right){VT1VT2}_{i-1,2}\left( t-1 \right)+\pi_{1,i}\lambda_{2,i}{VT1}_{i-1,2}\left( t-1 \right)+\pi_{4,i}\left( 1-d_{7}/2 \right)\lambda_{1,i}{VT2}_{i-1,2}\left( t-1 \right)+\rho_{i-1}{ALL}_{i-1,2}\left( t-1 \right)+\varphi_{0,i(if age<1)}\left( t-1 \right){VT1VT2}_{i-1,1}\left( t-1 \right)+\omega{VT1VT2}_{i-1,3}\left( t-1 \right),$$

$${VT1NVT}_{i,2}\left( t \right)=\left( 1-{2\rho}_{i-1}-{\pi_{8,i}\lambda}_{2,i}\left( t-1 \right)-\varphi_{1,i}(t-1)-\varphi_{2,i}(t-1) \right){VT1NVT}_{i-1,2}\left( t-1 \right)+\pi_{2,i}\lambda_{3,i}\left( t-1 \right){VT1}_{i-1,2}\left( t-1 \right)+\pi_{5,i}\left( 1-d_{7}/2 \right)\lambda_{1,i}{\left( t-1 \right)NVT}_{i-1,2}\left( t-1 \right)+\rho_{i-1}{ALL}_{i-1,2}\left( t-1 \right)+\varphi_{0,i(if age<1)}\left( t-1 \right){VT1NVT}_{i-1,1}\left( t-1 \right)+\omega{VT1NVT}_{i-1,3}\left( t-1 \right),$$

$${VT2NVT}_{i,2}\left( t \right)=\left( 1-{2\rho}_{i-1}-{\pi_{9,i}\left( 1-d_{7}/2 \right)\lambda}_{1,i}\left( t-1 \right)-\varphi_{1,i}(t-1)-\varphi_{2,i}(t-1) \right){VT2NVT}_{i-1,2}\left( t-1 \right)+\pi_{3,i}\lambda_{3,i}{VT1}_{i-1,2}\left( t-1 \right)+\pi_{6,i}\lambda_{2,i}N{VT}_{i-1,2}\left( t-1 \right)+\rho_{i-1}{ALL}_{i-1,2}\left( t-1 \right)+\varphi_{0,i(if age<1)}\left( t-1 \right){VT2NVT}_{i-1,1}\left( t-1 \right)+\omega{VT2NVT}_{i-1,3}\left( t-1 \right),$$

$${ALL}_{i,2}\left( t \right)=\left( 1-{3\rho}_{i-1}-\varphi_{1,i}(t-1)-\varphi_{2,i}(t-1) \right){ALL}_{i-1,2}\left( t-1 \right)+\pi_{7,i}\lambda_{3,i}\left( t-1 \right){VT1VT2}_{i-1,2}\left( t-1 \right) +\pi_{8,i}\lambda_{2,i}\left( t-1 \right)VT1N{VT}_{i-1,2}\left( t-1 \right)+\pi_{9,i}{\left( 1-d_{7}/2 \right)\lambda}_{1,i}\left( t-1 \right)VT2N{VT}_{i-1,2}\left( t-1 \right)+\varphi_{0,i(if age<1)}\left( t-1 \right){ALL}_{i-1,1}\left( t-1 \right)+\omega{ALL}_{i-1,3}\left( t-1 \right),$$

**PCV7 fully protected**:

$$S_{i,3}\left( t \right)=\left( 1-\left( 1-d_{7} \right)\lambda_{1,i}\left( t-1 \right)-\lambda_{2,i}\left( t-1 \right)-\lambda_{3,i}\left( t-1 \right)-\omega\right)S_{i-1,3}\left( t-1 \right)+\rho_{i-1}\left( {VT1}_{i-1,3}\left( t-1 \right)+{VT2}_{i-1,3}\left( t-1 \right)+{NVT}_{i-1,3}\left( t-1 \right) \right)+\varphi_{0,i(if age\geq1)}\left( t-1 \right)S_{i-1,1}\left( t-1 \right)+\varphi_{1,i}(t-1)\left( S_{i-1,2}\left( t-1 \right)+S_{i-1,4}\left( t-1 \right) \right),$$

$${VT1}_{i,3}\left( t \right)=\left( 1-\rho_{i-1}-\pi_{1,i}\lambda_{2,i}\left( t-1 \right)-{\pi_{2,i}\lambda}_{3,i}\left( t-1 \right)-\omega\right){VT1}_{i-1,3}\left( t-1 \right)+\rho_{i-1}\left( {VT1VT2}_{i-1,3}\left( t-1 \right)+VT1{NVT}_{i-1,3}\left( t-1 \right) \right)+\left( 1-d_{7} \right)\lambda_{1,i}\left( t-1 \right)S_{i-1,2}\left( t-1 \right)+\varphi_{0,i(if age\geq1)}\left( t-1 \right){VT1}_{i-1,1}\left( t-1 \right)+\varphi_{1,i}\left( t-1 \right)\left( {VT1}_{i-1,2}\left( t-1 \right)+{VT1}_{i-1,4}\left( t-1 \right) \right),$$

$${VT2}_{i,3}\left( t \right)=\left( 1-\rho_{i-1}-\pi_{4,i}\left( 1-d_{7} \right)\lambda_{1,i}\left( t-1 \right)-{\pi_{2,i}\lambda}_{3,i}\left( t-1 \right)-\omega\right){VT2}_{i-1,3}\left( t-1 \right)+\rho_{i-1}\left( {VT1VT2}_{i-1,3}\left( t-1 \right)+VT2{NVT}_{i-1,3}\left( t-1 \right) \right)+\lambda_{2,i}\left( t-1 \right)S_{i-1,2}\left( t-1 \right)+\varphi_{0,i(if age\geq1)}\left( t-1 \right){VT2}_{i-1,1}\left( t-1 \right)+\varphi_{1,i}\left( t-1 \right)\left( {VT2}_{i-1,2}\left( t-1 \right)+{VT2}_{i-1,4}\left( t-1 \right) \right),$$

$${NVT}_{i,3}\left( t \right)=\left( 1-\rho_{i-1}-\pi_{5,i}{\left( 1-d_{7} \right)\lambda}_{1,i}\left( t-1 \right)-{\pi_{6,i}\lambda}_{2,i}\left( t-1 \right)-\omega\right){NVT}_{i-1,3}\left( t-1 \right)+\rho_{i-1}\left( {VT1NVT}_{i-1,3}\left( t-1 \right)+VT2{NVT}_{i-1,3}\left( t-1 \right) \right)+\lambda_{3,i}\left( t-1 \right)S_{i-1,2}\left( t-1 \right)+\varphi_{0,i(if age\geq1)}\left( t-1 \right){NVT}_{i-1,1}\left( t-1 \right)+\varphi_{1,i}\left( t-1 \right)\left( {NVT}_{i-1,2}\left( t-1 \right)+{NVT}_{i-1,4}\left( t-1 \right) \right),$$

$${VT1VT2}_{i,3}\left( t \right)=\left( 1-{2\rho}_{i-1}-{\pi_{7,i}\lambda}_{3,i}\left( t-1 \right)-\omega\right){VT1VT2}_{i-1,3}\left( t-1 \right)+\pi_{1,i}\lambda_{2,i}{VT1}_{i-1,3}\left( t-1 \right)+\pi_{4,i}{\left( 1-d_{7} \right)\lambda}_{1,i}{VT2}_{i-1,3}\left( t-1 \right)+\rho_{i-1}{ALL}_{i-1,3}\left( t-1 \right)+\varphi_{0,i(if age\geq1)}\left( t-1 \right){VT1VT2}_{i-1,1}\left( t-1 \right)+\varphi_{1,i}\left( t-1 \right)\left( {VT1VT2}_{i-1,2}\left( t-1 \right)+{VT1VT2}_{i-1,4}\left( t-1 \right) \right),$$

$${VT1NVT}_{i,3}\left( t \right)=\left( 1-{2\rho}_{i-1}-{\pi_{8,i}\lambda}_{2,i}\left( t-1 \right)-\omega\right){VT1NVT}_{i-1,3}\left( t-1 \right)+\pi_{2,i}\lambda_{3,i}\left( t-1 \right){VT1}_{i-1,3}\left( t-1 \right)+\pi_{5,i}\left( 1-d_{7} \right)\lambda_{1,i}{\left( t-1 \right)NVT}_{i-1,3}\left( t-1 \right)+\rho_{i-1}{ALL}_{i-1,3}\left( t-1 \right)+\varphi_{0,i(if age<1)}\left( t-1 \right){VT1NVT}_{i-1,1}\left( t-1 \right)+\varphi_{1,i}\left( t-1 \right)\left( {VT1VT2}_{i-1,2}\left( t-1 \right)+{VT1VT2}_{i-1,4}\left( t-1 \right) \right),$$

$${VT2NVT}_{i,3}\left( t \right)=\left( 1-{2\rho}_{i-1}-{\pi_{9,i}\left( 1-d_{7}/2 \right)\lambda}_{1,i}\left( t-1 \right)-\omega\right){VT2NVT}_{i-1,3}\left( t-1 \right)+\pi_{3,i}\lambda_{3,i}{VT1}_{i-1,3}\left( t-1 \right)+\pi_{6,i}\lambda_{2,i}N{VT}_{i-1,3}\left( t-1 \right)+\rho_{i-1}{ALL}_{i-1,3}\left( t-1 \right)+\varphi_{0,i(if age\geq1)}\left( t-1 \right){VT2NVT}_{i-1,1}\left( t-1 \right)+\varphi_{1,i}\left( t-1 \right)\left( {VT2NVT}_{i-1,2}\left( t-1 \right)+{VT2NVT}_{i-1,4}\left( t-1 \right) \right),$$

$${ALL}_{i,3}\left( t \right)=\left( 1-{3\rho}_{i-1}-\omega\right){ALL}_{i-1,3}\left( t-1 \right)+\pi_{7,i}\lambda_{3,i}\left( t-1 \right){VT1VT2}_{i-1,3}\left( t-1 \right) +\pi_{8,i}\lambda_{2,i}\left( t-1 \right)VT1N{VT}_{i-1,3}\left( t-1 \right)+\pi_{9,i}{\left( 1-d_{7}/2 \right)\lambda}_{1,i}\left( t-1 \right)VT2N{VT}_{i-1,3}\left( t-1 \right)+\varphi_{0,i(if age\geq1)}\left( t-1 \right){ALL}_{i-1,1}\left( t-1 \right)+\varphi_{1,i}\left( t-1 \right)\left( {ALL}_{i-1,2}\left( t-1 \right)+{ALL}_{i-1,4}\left( t-1 \right) \right),$$

**PCV7 waned**:

$$S_{i,4}\left( t \right)=\left( 1-\lambda_{1,i}\left( t-1 \right)-\lambda_{2,i}\left( t-1 \right)-\lambda_{3,i}\left( t-1 \right)-\varphi_{1,i}(t-1)-\varphi_{2,i}\left( t-1 \right) \right)S_{i-1,4}\left( t-1 \right)+\rho_{i-1}\left( {VT1}_{i-1,4}\left( t-1 \right)+{VT2}_{i-1,4}\left( t-1 \right)+{NVT}_{i-1,4}\left( t-1 \right) \right)+\omega S_{i-1,2}\left( t-1 \right),$$

$${VT1}_{i,4}\left( t \right)=\left( 1-\rho_{i-1}-\pi_{1,i}\lambda_{2,i}\left( t-1 \right)-{\pi_{2,i}\lambda}_{3,i}\left( t-1 \right)-\varphi_{1,i}(t-1)-\varphi_{2,i}(t-1) \right){VT1}_{i-1,4}\left( t-1 \right)+\rho_{i-1}\left( {VT1VT2}_{i-1,4}\left( t-1 \right)+VT1{NVT}_{i-1,4}\left( t-1 \right) \right)+\lambda_{1,i}\left( t-1 \right)S_{i-1,4}\left( t-1 \right)+\omega{VT1}_{i-1,2}\left( t-1 \right),$$

$${VT2}_{i,4}\left( t \right)=\left( 1-\rho_{i-1}-\pi_{4,i}\lambda_{1,i}\left( t-1 \right)-{\pi_{2,i}\lambda}_{3,i}\left( t-1 \right)-\varphi_{1,i}(t-1)-\varphi_{2,i}\left( t-1 \right) \right){VT2}_{i-1,4}\left( t-1 \right)+\lambda_{2,i}\left( t-1 \right)S_{i-1,4}\left( t-1 \right)+\rho_{i-1}\left( {VT1VT2}_{i-1,4}\left( t-1 \right)+VT2{NVT}_{i-1,4}\left( t-1 \right) \right)+\omega{VT2}_{i-1,2}\left( t-1 \right),$$

$${NVT}_{i,4}\left( t \right)=\left( 1-\rho_{i-1}-\pi_{5,i}\lambda_{1,i}\left( t-1 \right)-{\pi_{6,i}\lambda}_{2,i}\left( t-1 \right)-\varphi_{1,i}(t-1)-\varphi_{2,i}\left( t-1 \right) \right){NVT}_{i-1,4}\left( t-1 \right)+\lambda_{3,i}\left( t-1 \right)S_{i-1,4}\left( t-1 \right)+\rho_{i-1}\left( {VT1NVT}_{i-1,4}\left( t-1 \right)+VT2{NVT}_{i-1,4}\left( t-1 \right) \right)+\omega{NVT}_{i-1,2}\left( t-1 \right),$$

$${VT1VT2}_{i,4}\left( t \right)=\left( 1-{2\rho}_{i-1}-{\pi_{7,i}\lambda}_{3,i}\left( t-1 \right)-\varphi_{1,i}(t-1)-\varphi_{2,i}(t-1) \right){VT1VT2}_{i-1,4}\left( t-1 \right)+\pi_{1,i}\lambda_{2,i}{VT1}_{i-1,4}\left( t-1 \right)+\pi_{4,i}\lambda_{1,i}{VT2}_{i-1,4}\left( t-1 \right)+\rho_{i-1}{ALL}_{i-1,4}\left( t-1 \right)+\omega{VT1VT2}_{i-1,2}\left( t-1 \right),$$

$${VT1NVT}_{i,4}\left( t \right)=\left( 1-{2\rho}_{i-1}-{\pi_{8,i}\lambda}_{2,i}\left( t-1 \right)-\varphi_{1,i}(t-1)-\varphi_{2,i}(t-1) \right){VT1NVT}_{i-1,4}\left( t-1 \right)+\pi_{2,i}\lambda_{3,i}\left( t-1 \right){VT1}_{i-1,4}\left( t-1 \right)+\pi_{5,i}\lambda_{1,i}{\left( t-1 \right)NVT}_{i-1,4}\left( t-1 \right)+\rho_{i-1}{ALL}_{i-1,4}\left( t-1 \right)+\omega{VT1NVT}_{i-1,2}\left( t-1 \right),$$

$${VT2NVT}_{i,4}\left( t \right)=\left( 1-{2\rho}_{i-1}-{\pi_{9,i}\lambda}_{1,i}\left( t-1 \right)-\varphi_{1,i}(t-1)-\varphi_{2,i}(t-1) \right){VT2NVT}_{i-1,4}\left( t-1 \right)+\pi_{3,i}\lambda_{3,i}{VT1}_{i-1,4}\left( t-1 \right)+\pi_{6,i}\lambda_{2,i}N{VT}_{i-1,4}\left( t-1 \right)+\rho_{i-1}{ALL}_{i-1,4}\left( t-1 \right)+\omega{VT2NVT}_{i-1,2}\left( t-1 \right),$$

$${ALL}_{i,4}\left( t \right)=\left( 1-{3\rho}_{i-1}-\varphi_{1,i}(t-1)-\varphi_{2,i}(t-1) \right){ALL}_{i-1,4}\left( t-1 \right)+\pi_{7,i}\lambda_{3,i}\left( t-1 \right){VT1VT2}_{i-1,4}\left( t-1 \right) +\pi_{8,i}\lambda_{2,i}\left( t-1 \right)VT1N{VT}_{i-1,4}\left( t-1 \right)+\pi_{9,i}\lambda_{1,i}\left( t-1 \right)VT2N{VT}_{i-1,4}\left( t-1 \right)+\omega{ALL}_{i-1,2}\left( t-1 \right),$$

**PCV13 partially protected**:

$$S_{i,5}\left( t \right)=\left( 1-\left( 1-d_{7}/2 \right)\lambda_{1,i}\left( t-1 \right)-{\left( 1-d_{13}/2 \right)\lambda}_{2,i}\left( t-1 \right)-\lambda_{3,i}\left( t-1 \right)-\varphi_{1,i}(t-1)-\varphi_{2,i}\left( t-1 \right) \right)S_{i-1,5}\left( t-1 \right)+\rho_{i-1}\left( {VT1}_{i-1,5}\left( t-1 \right)+{VT2}_{i-1,5}\left( t-1 \right)+{NVT}_{i-1,5}\left( t-1 \right) \right)+\varphi_{0,i(if age<1)}\left( t-1 \right)S_{i-1,1}\left( t-1 \right)+\omega S_{i-1,6}\left( t-1 \right),$$

$${VT1}_{i,5}\left( t \right)=\left( 1-\rho_{i-1}-\pi_{1,i}{\left( 1-d_{13}/2 \right)\lambda}_{2,i}\left( t-1 \right)-{\pi_{2,i}\lambda}_{3,i}\left( t-1 \right)-\varphi_{1,i}(t-1)-\varphi_{2,i}(t-1) \right){VT1}_{i-1,5}\left( t-1 \right)+\rho_{i-1}\left( {VT1VT2}_{i-1,5}\left( t-1 \right)+VT1{NVT}_{i-1,5}\left( t-1 \right) \right)+\left( 1-d_{7}/2 \right)\lambda_{1,i}\left( t-1 \right)S_{i-1,5}\left( t-1 \right)+\varphi_{0,i(if age<1)}\left( t-1 \right){VT1}_{i-1,1}\left( t-1 \right)+\omega{VT1}_{i-1,6}\left( t-1 \right),$$

$${VT2}_{i,5}\left( t \right)=\left( 1-\rho_{i-1}-\pi_{4,i}\left( 1-d_{7}/2 \right)\lambda_{1,i}\left( t-1 \right)-{\pi_{2,i}\lambda}_{3,i}\left( t-1 \right)-\varphi_{1,i}(t-1)-\varphi_{2,i}\left( t-1 \right) \right){VT2}_{i-1,5}\left( t-1 \right)+{\left( 1-d_{13}/2 \right)\lambda}_{2,i}\left( t-1 \right)S_{i-1,5}\left( t-1 \right)+\rho_{i-1}\left( {VT1VT2}_{i-1,5}\left( t-1 \right)+VT2{NVT}_{i-1,5}\left( t-1 \right) \right)+\varphi_{0,i(if age<1)}\left( t-1 \right){VT2}_{i-1,1}\left( t-1 \right)+\omega{VT2}_{i-1,6}\left( t-1 \right),$$

$${NVT}_{i,5}\left( t \right)=\left( 1-\rho_{i-1}-\pi_{5,i}{\left( 1-d_{7}/2 \right)\lambda}_{1,i}\left( t-1 \right)-{\pi_{6,i}\left( 1-d_{13}/2 \right)\lambda}_{2,i}\left( t-1 \right)-\varphi_{1,i}(t-1)-\varphi_{2,i}\left( t-1 \right) \right){NVT}_{i-1,5}\left( t-1 \right)+\lambda_{3,i}\left( t-1 \right)S_{i-1,5}\left( t-1 \right)+\rho_{i-1}\left( {VT1NVT}_{i-1,5}\left( t-1 \right)+VT2{NVT}_{i-1,5}\left( t-1 \right) \right)+\varphi_{0,i(if age<1)}\left( t-1 \right){NVT}_{i-1,1}\left( t-1 \right)+\omega{NVT}_{i-1,6}\left( t-1 \right),$$

$${VT1VT2}_{i,5}\left( t \right)=\left( 1-{2\rho}_{i-1}-{\pi_{7,i}\lambda}_{3,i}\left( t-1 \right)-\varphi_{1,i}(t-1)-\varphi_{2,i}(t-1) \right){VT1VT2}_{i-1,5}\left( t-1 \right)+\pi_{1,i}\left( 1-d_{13}/2 \right)\lambda_{2,i}{VT1}_{i-1,5}\left( t-1 \right)+\pi_{4,i}\left( 1-d_{7}/2 \right)\lambda_{1,i}{VT2}_{i-1,5}\left( t-1 \right)+\rho_{i-1}{ALL}_{i-1,5}\left( t-1 \right)+\varphi_{0,i(if age<1)}\left( t-1 \right){VT1VT2}_{i-1,1}\left( t-1 \right)+\omega{VT1VT2}_{i-1,6}\left( t-1 \right),$$

$${VT1NVT}_{i,5}\left( t \right)=\left( 1-{2\rho}_{i-1}-{\pi_{8,i}\left( 1-d_{13}/2 \right)\lambda}_{2,i}\left( t-1 \right)-\varphi_{1,i}(t-1)-\varphi_{2,i}(t-1) \right){VT1NVT}_{i-1,5}\left( t-1 \right)+\pi_{2,i}\lambda_{3,i}\left( t-1 \right){VT1}_{i-1,5}\left( t-1 \right)+\pi_{5,i}\left( 1-d_{7}/2 \right)\lambda_{1,i}{\left( t-1 \right)NVT}_{i-1,5}\left( t-1 \right)+\rho_{i-1}{ALL}_{i-1,5}\left( t-1 \right)+\varphi_{0,i(if age<1)}\left( t-1 \right){VT1NVT}_{i-1,1}\left( t-1 \right)+\omega{VT1NVT}_{i-1,6}\left( t-1 \right),$$

$${VT2NVT}_{i,5}\left( t \right)=\left( 1-{2\rho}_{i-1}-{\pi_{9,i}\left( 1-d_{7}/2 \right)\lambda}_{1,i}\left( t-1 \right)-\varphi_{1,i}(t-1)-\varphi_{2,i}(t-1) \right){VT2NVT}_{i-1,5}\left( t-1 \right)+\pi_{3,i}\lambda_{3,i}{VT1}_{i-1,5}\left( t-1 \right)+\pi_{6,i}{\left( 1-d_{13}/2 \right)\lambda}_{2,i}N{VT}_{i-1,5}\left( t-1 \right)+\rho_{i-1}{ALL}_{i-1,5}\left( t-1 \right)+\varphi_{0,i(if age<1)}\left( t-1 \right){VT2NVT}_{i-1,1}\left( t-1 \right)+\omega{VT2NVT}_{i-1,6}\left( t-1 \right),$$

$${ALL}_{i,5}\left( t \right)=\left( 1-{3\rho}_{i-1}-\varphi_{1,i}(t-1)-\varphi_{2,i}(t-1) \right){ALL}_{i-1,5}\left( t-1 \right)+\pi_{7,i}\lambda_{3,i}\left( t-1 \right){VT1VT2}_{i-1,5}\left( t-1 \right) +\pi_{8,i}{\left( 1-d_{13}/2 \right)\lambda}_{2,i}\left( t-1 \right)VT1N{VT}_{i-1,5}\left( t-1 \right)+\pi_{9,i}{\left( 1-d_{7}/2 \right)\lambda}_{1,i}\left( t-1 \right)VT2N{VT}_{i-1,5}\left( t-1 \right)+\varphi_{0,i(if age<1)}\left( t-1 \right){ALL}_{i-1,1}\left( t-1 \right)+\omega{ALL}_{i-1,6}\left( t-1 \right),$$

**PCV13 fully protected:**

$$S_{i,6}\left( t \right)=\left( 1-\left( 1-d_{7} \right)\lambda_{1,i}\left( t-1 \right)-{\left( 1-d_{13} \right)\lambda}_{2,i}\left( t-1 \right)-\lambda_{3,i}\left( t-1 \right)-\omega\right)S_{i-1,6}\left( t-1 \right)+\rho_{i-1}\left( {VT1}_{i-1,6}\left( t-1 \right)+{VT2}_{i-1,6}\left( t-1 \right)+{NVT}_{i-1,6}\left( t-1 \right) \right)+\varphi_{0,i(if age\geq1)}\left( t-1 \right)S_{i-1,1}\left( t-1 \right)+\varphi_{1,i}(t-1)\left( S_{i-1,2}\left( t-1 \right)+S_{i-1,4}\left( t-1 \right)+S_{i-1,5}\left( t-1 \right)+S_{i-1,7}\left( t-1 \right) \right),$$

$${VT1}_{i,6}\left( t \right)=\left( 1-\rho_{i-1}-\pi_{1,i}{\left( 1-d_{13} \right)\lambda}_{2,i}\left( t-1 \right)-{\pi_{2,i}\lambda}_{3,i}\left( t-1 \right)-\omega\right){VT1}_{i-1,6}\left( t-1 \right)+\rho_{i-1}\left( {VT1VT2}_{i-1,6}\left( t-1 \right)+VT1{NVT}_{i-1,6}\left( t-1 \right) \right)+\left( 1-d_{7} \right)\lambda_{1,i}\left( t-1 \right)S_{i-1,6}\left( t-1 \right)+\varphi_{0,i(if age\geq1)}\left( t-1 \right){VT1}_{i-1,1}\left( t-1 \right)+\varphi_{1,i}\left( t-1 \right)\left( {VT1}_{i-1,2}\left( t-1 \right)+{VT1}_{i-1,4}\left( t-1 \right)+{VT1}_{i-1,5}\left( t-1 \right)+{VT1}_{i-1,7}\left( t-1 \right) \right),$$

$${VT2}_{i,6}\left( t \right)=\left( 1-\rho_{i-1}-\pi_{4,i}\left( 1-d_{7} \right)\lambda_{1,i}\left( t-1 \right)-{\pi_{2,i}\lambda}_{3,i}\left( t-1 \right)-\omega\right){VT2}_{i-1,6}\left( t-1 \right)+\rho_{i-1}\left( {VT1VT2}_{i-1,6}\left( t-1 \right)+VT2{NVT}_{i-1,6}\left( t-1 \right) \right)+{\left( 1-d_{13} \right)\lambda}_{2,i}\left( t-1 \right)S_{i-1,6}\left( t-1 \right)+\varphi_{0,i(if age\geq1)}\left( t-1 \right){VT2}_{i-1,1}\left( t-1 \right)+\varphi_{1,i}\left( t-1 \right)\left( {VT2}_{i-1,2}\left( t-1 \right)+{VT2}_{i-1,4}\left( t-1 \right)+{VT2}_{i-1,5}\left( t-1 \right)+{VT2}_{i-1,7}\left( t-1 \right) \right),$$

$${NVT}_{i,6}\left( t \right)=\left( 1-\rho_{i-1}-\pi_{5,i}{\left( 1-d_{7} \right)\lambda}_{1,i}\left( t-1 \right)-{\pi_{6,i}\left( 1-d_{13} \right)\lambda}_{2,i}\left( t-1 \right)-\omega\right){NVT}_{i-1,6}\left( t-1 \right)+\rho_{i-1}\left( {VT1NVT}_{i-1,6}\left( t-1 \right)+VT2{NVT}_{i-1,6}\left( t-1 \right) \right)+\lambda_{3,i}\left( t-1 \right)S_{i-1,6}\left( t-1 \right)+\varphi_{0,i(if age\geq1)}\left( t-1 \right){NVT}_{i-1,1}\left( t-1 \right)+\varphi_{1,i}\left( t-1 \right)\left( {NVT}_{i-1,2}\left( t-1 \right)+{NVT}_{i-1,4}\left( t-1 \right)+{NVT}_{i-1,5}\left( t-1 \right)+{NVT}_{i-1,7}\left( t-1 \right) \right),$$

$${VT1VT2}_{i,6}\left( t \right)=\left( 1-{2\rho}_{i-1}-{\pi_{7,i}\lambda}_{3,i}\left( t-1 \right)-\omega\right){VT1VT2}_{i-1,6}\left( t-1 \right)+\pi_{1,i}{\left( 1-d_{13} \right)\lambda}_{2,i}{VT1}_{i-1,6}\left( t-1 \right)+\pi_{4,i}{\left( 1-d_{7} \right)\lambda}_{1,i}{VT2}_{i-1,6}\left( t-1 \right)+\rho_{i-1}{ALL}_{i-1,6}\left( t-1 \right)+\varphi_{0,i(if age\geq1)}\left( t-1 \right){VT1VT2}_{i-1,1}\left( t-1 \right)+\varphi_{1,i}\left( t-1 \right)\left( {VT1VT2}_{i-1,2}\left( t-1 \right)+{VT1VT2}_{i-1,4}\left( t-1 \right)+{VT1VT2}_{i-1,5}\left( t-1 \right)+{VT1VT2}_{i-1,7}\left( t-1 \right) \right),$$

$${VT1NVT}_{i,6}\left( t \right)=\left( 1-{2\rho}_{i-1}-{\pi_{8,i}\left( 1-d_{13} \right)\lambda}_{2,i}\left( t-1 \right)-\omega\right){VT1NVT}_{i-1,6}\left( t-1 \right)+\pi_{2,i}\lambda_{3,i}\left( t-1 \right){VT1}_{i-1,6}\left( t-1 \right)+\pi_{5,i}\left( 1-d_{7} \right)\lambda_{1,i}{\left( t-1 \right)NVT}_{i-1,6}\left( t-1 \right)+\rho_{i-1}{ALL}_{i-1,6}\left( t-1 \right)+\varphi_{0,i(if age<1)}\left( t-1 \right){VT1NVT}_{i-1,1}\left( t-1 \right)+\varphi_{1,i}\left( t-1 \right)\left( {VT1VT2}_{i-1,2}\left( t-1 \right)+{VT1VT2}_{i-1,4}\left( t-1 \right)+{VT1VT2}_{i-1,5}\left( t-1 \right)+{VT1VT2}_{i-1,7}\left( t-1 \right) \right),$$

$${VT2NVT}_{i,6}\left( t \right)=\left( 1-{2\rho}_{i-1}-{\pi_{9,i}\left( 1-d_{7}/2 \right)\lambda}_{1,i}\left( t-1 \right)-\omega\right){VT2NVT}_{i-1,6}\left( t-1 \right)+\pi_{3,i}\lambda_{3,i}{VT1}_{i-1,6}\left( t-1 \right)+\pi_{6,i}\left( 1-d_{13} \right)\lambda_{2,i}N{VT}_{i-1,6}\left( t-1 \right)+\rho_{i-1}{ALL}_{i-1,6}\left( t-1 \right)+\varphi_{0,i(if age\geq1)}\left( t-1 \right){VT2NVT}_{i-1,1}\left( t-1 \right)+\varphi_{1,i}\left( t-1 \right)\left( {VT2NVT}_{i-1,2}\left( t-1 \right)+{VT2NVT}_{i-1,4}\left( t-1 \right)+{VT2NVT}_{i-1,5}\left( t-1 \right)+{VT2NVT}_{i-1,7}\left( t-1 \right) \right),$$

$${ALL}_{i,6}\left( t \right)=\left( 1-{3\rho}_{i-1}-\omega\right){ALL}_{i-1,3}\left( t-1 \right)+\pi_{7,i}\lambda_{3,i}\left( t-1 \right){VT1VT2}_{i-1,6}\left( t-1 \right) +\pi_{8,i}\left( 1-d_{13} \right)\lambda_{2,i}\left( t-1 \right)VT1N{VT}_{i-1,6}\left( t-1 \right)+\pi_{9,i}{\left( 1-d_{7}/2 \right)\lambda}_{1,i}\left( t-1 \right)VT2N{VT}_{i-1,6}\left( t-1 \right)+\varphi_{0,i(if age\geq1)}\left( t-1 \right){ALL}_{i-1,1}\left( t-1 \right)+\varphi_{1,i}\left( t-1 \right)\left( {ALL}_{i-1,2}\left( t-1 \right)+{ALL}_{i-1,4}\left( t-1 \right)+{ALL}_{i-1,5}\left( t-1 \right)+{ALL}_{i-1,7}\left( t-1 \right) \right),$$

**PCV13 waned:**

$$S_{i,7}\left( t \right)=\left( 1-\lambda_{1,i}\left( t-1 \right)-\lambda_{2,i}\left( t-1 \right)-\lambda_{3,i}\left( t-1 \right)-\varphi_{1,i}(t-1)-\varphi_{2,i}\left( t-1 \right) \right)S_{i-1,7}\left( t-1 \right)+\rho_{i-1}\left( {VT1}_{i-1,7}\left( t-1 \right)+{VT2}_{i-1,7}\left( t-1 \right)+{NVT}_{i-1,7}\left( t-1 \right) \right)+\omega S_{i-1,5}\left( t-1 \right),$$

$${VT1}_{i,7}\left( t \right)=\left( 1-\rho_{i-1}-\pi_{1,i}\lambda_{2,i}\left( t-1 \right)-{\pi_{2,i}\lambda}_{3,i}\left( t-1 \right)-\varphi_{1,i}(t-1)-\varphi_{2,i}(t-1) \right){VT1}_{i-1,7}\left( t-1 \right)+\rho_{i-1}\left( {VT1VT2}_{i-1,7}\left( t-1 \right)+VT1{NVT}_{i-1,7}\left( t-1 \right) \right)+\lambda_{1,i}\left( t-1 \right)S_{i-1,7}\left( t-1 \right)+\omega{VT1}_{i-1,5}\left( t-1 \right),$$

$${VT2}_{i,7}\left( t \right)=\left( 1-\rho_{i-1}-\pi_{4,i}\lambda_{1,i}\left( t-1 \right)-{\pi_{2,i}\lambda}_{3,i}\left( t-1 \right)-\varphi_{1,i}(t-1)-\varphi_{2,i}\left( t-1 \right) \right){VT2}_{i-1,7}\left( t-1 \right)+\lambda_{2,i}\left( t-1 \right)S_{i-1,7}\left( t-1 \right)+\rho_{i-1}\left( {VT1VT2}_{i-1,7}\left( t-1 \right)+VT2{NVT}_{i-1,7}\left( t-1 \right) \right)+\omega{VT2}_{i-1,5}\left( t-1 \right),$$

$${NVT}_{i,7}\left( t \right)=\left( 1-\rho_{i-1}-\pi_{5,i}\lambda_{1,i}\left( t-1 \right)-{\pi_{6,i}\lambda}_{2,i}\left( t-1 \right)-\varphi_{1,i}(t-1)-\varphi_{2,i}\left( t-1 \right) \right){NVT}_{i-1,7}\left( t-1 \right)+\lambda_{3,i}\left( t-1 \right)S_{i-1,7}\left( t-1 \right)+\rho_{i-1}\left( {VT1NVT}_{i-1,7}\left( t-1 \right)+VT2{NVT}_{i-1,7}\left( t-1 \right) \right)+\omega{NVT}_{i-1,5}\left( t-1 \right),$$

$${VT1VT2}_{i,7}\left( t \right)=\left( 1-{2\rho}_{i-1}-{\pi_{7,i}\lambda}_{3,i}\left( t-1 \right)-\varphi_{1,i}(t-1)-\varphi_{2,i}(t-1) \right){VT1VT2}_{i-1,7}\left( t-1 \right)+\pi_{1,i}\lambda_{2,i}{VT1}_{i-1,7}\left( t-1 \right)+\pi_{4,i}\lambda_{1,i}{VT2}_{i-1,7}\left( t-1 \right)+\rho_{i-1}{ALL}_{i-1,7}\left( t-1 \right)+\omega{VT1VT2}_{i-1,5}\left( t-1 \right),$$

$${VT1NVT}_{i,7}\left( t \right)=\left( 1-{2\rho}_{i-1}-{\pi_{8,i}\lambda}_{2,i}\left( t-1 \right)-\varphi_{1,i}(t-1)-\varphi_{2,i}(t-1) \right){VT1NVT}_{i-1,7}\left( t-1 \right)+\pi_{2,i}\lambda_{3,i}\left( t-1 \right){VT1}_{i-1,7}\left( t-1 \right)+\pi_{5,i}\lambda_{1,i}{\left( t-1 \right)NVT}_{i-1,7}\left( t-1 \right)+\rho_{i-1}{ALL}_{i-1,7}\left( t-1 \right)+\omega{VT1NVT}_{i-1,5}\left( t-1 \right),$$

$${VT2NVT}_{i,7}\left( t \right)=\left( 1-{2\rho}_{i-1}-{\pi_{9,i}\lambda}_{1,i}\left( t-1 \right)-\varphi_{1,i}(t-1)-\varphi_{2,i}(t-1) \right){VT2NVT}_{i-1,7}\left( t-1 \right)+\pi_{3,i}\lambda_{3,i}{VT1}_{i-1,7}\left( t-1 \right)+\pi_{6,i}\lambda_{2,i}N{VT}_{i-1,7}\left( t-1 \right)+\rho_{i-1}{ALL}_{i-1,7}\left( t-1 \right)+\omega{VT2NVT}_{i-1,5}\left( t-1 \right),$$

$${ALL}_{i,7}\left( t \right)=\left( 1-{3\rho}_{i-1}-\varphi_{1,i}(t-1)-\varphi_{2,i}(t-1) \right){ALL}_{i-1,7}\left( t-1 \right)+\pi_{7,i}\lambda_{3,i}\left( t-1 \right){VT1VT2}_{i-1,7}\left( t-1 \right) +\pi_{8,i}\lambda_{2,i}\left( t-1 \right)VT1N{VT}_{i-1,7}\left( t-1 \right)+\pi_{9,i}\lambda_{1,i}\left( t-1 \right)VT2N{VT}_{i-1,7}\left( t-1 \right)+\omega{ALL}_{i-1,5}\left( t-1 \right),$$

for $i=2,\ldots,4800$ age cohorts (48 cohorts for each annual age cohort comprising 100 year cohorts between 0y and 99y) , where the initial values of Unvaccinated group is obtained from the pre-vaccination equilibrium from the static model result. The movement between vaccine protected groups depends on the monthly vaccine uptake and vaccine protection waning according to the duration of vaccine protection. $\pi$ is a reduction parameter on the FOI, $\lambda,$ which is 1- Competition parameter between serogroups, $\varphi$s are monthly vaccination rates for each dose (two primary and booster doses), d7 and d13 are reduction in FOIs due to PCV7 and PCV13 against acquisition of VT1 and VT2, $\omega$ is a waning parameter (1/duration of vaccine protection), and $\rho$ is a clearance rate, 1/ duration of colonisation.

The Nelder-Mead method finds the set of model parameters with the maximum Poisson likelihood:

$LogLikelihood (Model|data)= \sum_{Y=2005}^{2015} \sum_{Sero=1}^{3} \sum_{Age=1}^{6} \left( {IPDData}_{Y,Sero,Age} log({IPDModel}_{Y,Sero,Age})-{IPDModel}_{Y,Sero,Age} \right)$

for three serogroups and six age groups between 2005/06 and 2015/16.
